# AMBIsome Therapy Induction OptimisatioN (AMBITION): High Dose AmBisome for Cryptococcal Meningitis Induction Therapy in sub-Saharan Africa: Study Protocol for a Phase 3 Randomised Controlled Non-Inferiority Trial

**DOI:** 10.1186/s13063-018-3026-4

**Published:** 2018-11-23

**Authors:** David S. Lawrence, Nabila Youssouf, Síle L. F. Molloy, Alexandre Alanio, Melanie Alufandika, David R. Boulware, Timothée Boyer-Chammard, Tao Chen, Francoise Dromer, Admire Hlupeni, William Hope, Mina C. Hosseinipour, Cecilia Kanyama, Oliver Lortholary, Angela Loyse, David B. Meya, Mosepele Mosepele, Conrad Muzoora, Henry C. Mwandumba, Chiratidzo E. Ndhlovu, Louis Niessen, Charlotte Schutz, Katharine E. Stott, Duolao Wang, David G. Lalloo, Graeme Meintjes, Shabbar Jaffar, Thomas S. Harrison, Joseph N. Jarvis

**Affiliations:** 1 Department of Clinical Research, Faculty of Infectious and Tropical Diseases, London School of Hygiene and Tropical Medicine, London, UK; 2 Botswana-Harvard AIDS Institute Partnership, Gaborone, Botswana; 3 Research Centre for Infection and Immunity, St George’s University of London, London, UK; 4 Molecular Mycology Unit and National Reference Centre for Invasive Mycoses, Institut Pasteur, Paris, France; 5 Malawi-Liverpool-Wellcome Trust Clinical Research Programme, University of Malawi College of Medicine, Blantyre, Malawi; 6 Infectious Diseases Institute, College of Health Sciences, Makerere University, Kampala, Uganda; 7 Department of Medicine, University of Minnesota, Minneapolis, MN USA; 8 Department of Clinical Sciences and International Public Health, Liverpool School of Tropical Medicine, Liverpool, UK; 9 Department of Medicine, University of Zimbabwe College of Health Sciences, Parirenyatwa Hospital, Harare, Zimbabwe; 10 Department of Molecular and Clinical Pharmacology, University of Liverpool, Liverpool, UK; 11 Lilongwe Medical Relief Trust (UNC Project), Lilongwe, Malawi; 12 Department of Internal Medicine, University of Botswana, Gaborone, Botswana; 13 Wellcome Centre for Infectious Diseases Research in Africa (CIDRI-Africa), Institute of Infectious Disease and Molecular Medicine, and Department of Medicine, University of Cape Town, Cape Town, South Africa

**Keywords:** Cryptococcal meningitis, HIV, AmBisome, Amphotericin B, Fluconazole, Flucytosine, Clinical trial

## Abstract

**Background:**

Cryptococcal meningitis (CM) is a major cause of mortality in HIV programmes in Africa despite increasing access to antiretroviral therapy (ART). Mortality is driven in part by limited availability of amphotericin-based treatment, drug-induced toxicities of amphotericin B deoxycholate and prolonged hospital admissions. A single, high-dose of liposomal amphotericin (L-AmB, Ambisome) on a fluconazole backbone has been reported as non-inferior to 14 days of standard dose L-AmB in reducing fungal burden. This trial examines whether single, high-dose L-AmB given with high-dose fluconazole and flucytosine is non-inferior to a seven-day course of amphotericin B deoxycholate plus flucytosine (the current World Health Organization [WHO] recommended treatment regimen).

**Methods:**

An open-label phase III randomised controlled non-inferiority trial conducted in five countries in sub-Saharan Africa: Botswana, Malawi, South Africa, Uganda and Zimbabwe. The trial will compare CM induction therapy with (1) a single dose (10 mg/kg) of L-AmB given with 14 days of fluconazole (1200 mg/day) and flucytosine (100 mg/kg/day) to (2) seven days amphotericin B deoxycholate (1 mg/kg/day) given alongside seven days of flucytosine (100 mg/kg/day) followed by seven days of fluconazole (1200 mg/day). The primary endpoint is all-cause mortality at ten weeks with a non-inferiority margin of 10% and 90% power. Secondary endpoints are early fungicidal activity, proportion of grade III/IV adverse events, pharmacokinetic parameters and pharmacokinetic/pharmacodynamic associations, health service costs, all-cause mortality within the first two and four weeks, all-cause mortality within the first ten weeks (superiority analysis) and rates of CM relapse, immune reconstitution inflammatory syndrome and disability at ten weeks. A total of 850 patients aged ≥ 18 years with a first episode of HIV-associated CM will be enrolled (425 randomised to each arm). All patients will be followed for 16 weeks. All patients will receive consolidation therapy with fluconazole 800 mg/day to complete ten weeks of treatment, followed by fluconazole maintenance and ART as per local guidance.

**Discussion:**

A safe, sustainable and easy to administer regimen of L-AmB that is non-inferior to seven days of daily amphotericin B deoxycholate therapy may reduce the number of adverse events seen in patients treated with amphotericin B deoxycholate and shorten hospital admissions, providing a highly favourable and implementable alternative to the current WHO recommended first-line treatment.

**Trial registration:**

ISRCTN, ISRCTN72509687. Registered on 13 July 2017.

**Electronic supplementary material:**

The online version of this article (10.1186/s13063-018-3026-4) contains supplementary material, which is available to authorized users.

## Background

Early mortality among people initiating HIV treatment in Africa is considerably higher than in high-income countries [1–4]. Despite antiretroviral therapy (ART) roll-out, approximately half of HIV-infected individuals in sub-Saharan Africa are not on ART and about one-third still present for care with very low CD4 counts. The incidence of opportunistic co-infections such as CM in this group is high [5] and CM remains the most common cause of adult meningitis in much of Africa [6]. As a result, cryptococcal meningitis (CM) is a major cause of mortality in HIV-infected patients in Africa and is associated with 10–20% of all HIV-related deaths [7]. Furthermore, the number of CM cases remains high despite increased ART access; they now include both ART-naïve and ART-experienced patients, with half of patients diagnosed with CM having had prior exposure to ART but with persisting low CD4 counts due to non-adherence and/or ART failure [8–10]. The poor outcomes reported using currently available antifungal therapy in African centres are a critical driver of this high mortality. Mortality using amphotericin B deoxycholate-based therapy in Africa, even in clinical trial settings, remains in the region of 35–45% [10–13]. Amphotericin B deoxycholate therapy requires hospitalisation for at least seven days and its toxicity profile requires costly laboratory monitoring. The average hospitalisation cost for CM treated with amphotericin B deoxycholate is USD 800–1000 in Zimbabwe where the annual per capita gross domestic product is < USD 1000. Many clinical centres in sub-Saharan Africa lack access to reliable laboratory monitoring and have limited nursing capacity making safe administration of conventional amphotericin B deoxycholate difficult or impossible. Consequently, amphotericin B deoxycholate therapy is often not available in Africa. Fluconazole, the oral alternative widely used in Africa, is much less rapidly fungicidal than amphotericin-B, even at a dosage of up to 1200 mg/day, and mortality at ten weeks is 50–60% [14, 15]. Given the HIV prevalence and incidence in Southern and East Africa, inadequate ART coverage, suboptimal monitoring of individuals on ART leading to treatment failure and limited access to screening and pre-emptive treatment for CM, CM will remain a major cause of morbidity and mortality in the region for the foreseeable future. New treatment strategies are urgently needed.

Until recently, World Health Organization (WHO) treatment guidelines recommended a 14-day course of amphotericin B deoxycholate-based treatment for CM induction therapy. The recently completed phase III ACTA trial showed that patients receiving a short, seven-day course of amphotericin B deoxycholate plus flucytosine had lower mortality at ten weeks (24%, 95% confidence interval [CI]: 16–32) compared to patients receiving 14-day course of amphotericin B plus flucytosine (38%, 95% CI: 29–47, unadjusted hazard ratio [HR] 0.56, 95% CI: 0.35–0.91) [10]. The trial also confirmed that flucytosine (5FC) is a significantly superior partner drug for amphotericin B-based treatments compared with fluconazole, leading to a substantial mortality reduction of 38% (95% CI: 16–55, *p* = 0.002). As a consequence, the WHO guidelines were revised and now recommend first-line treatment with seven days of amphotericin B deoxycholate and flucytosine 100 mg/kg/day followed by seven days of fluconazole 1200 mg/day. In settings where flucytosine is unavailable, which reflects most settings in Africa, the guidelines continue to recommend 14 days of amphotericin B deoxycholate with fluconazole [16].

A newer lipid-based formulation of amphotericin B deoxycholate (L-AmB or AmBisome©) is particularly suited for use in short-course yet highly effective induction treatment for HIV-associated CM, due to: (1) the potential for high dosing made possible by the lower rates of drug-induced toxicity; and (2) the long tissue half-life. In the context of HIV-associated CM, 14-day courses of conventional amphotericin B deoxycholate are associated with an average drop in haemoglobin of 2.3 g/dL and a mean increase in creatinine of 73% [17]. Even at high doses, L-AmB is associated with significantly less nephrotoxicity and anaemia as well as lower rates of infusion reactions than conventional amphotericin B deoxycholate [18]. The long tissue half-life of L-AmB following high-dose administration in patients is well-established [19–22], as is its effective penetration into brain tissue [23]. The concept of single or intermittent dosing with very high doses is also established in both prophylaxis in haematology patients and treatment of visceral leishmaniasis in lower- and middle-income countries [24]. Single doses of up to 15 mg/kg have been safely given; doses of 10 mg/kg are routinely given with demonstration of efficacy for treatment of visceral leishmaniasis and invasive fungal infections [24, 25]. Pharmacokinetic data from animal models [20] and humans [19] suggest that increasing L-AmB dosing from the currently recommended 3–4 mg/kg may lead to improved outcomes and, as with standard amphotericin B, that intermittent dosing regimens may be as effective as daily therapy [20]. Although L-AmB is recommended as treatment for HIV-associated CM in several national guidelines, optimal dosing is unknown and the strategy of short-course high dosing of L-AmB has not yet been tested in a phase III clinical trial [18].

**Fig. 1:**
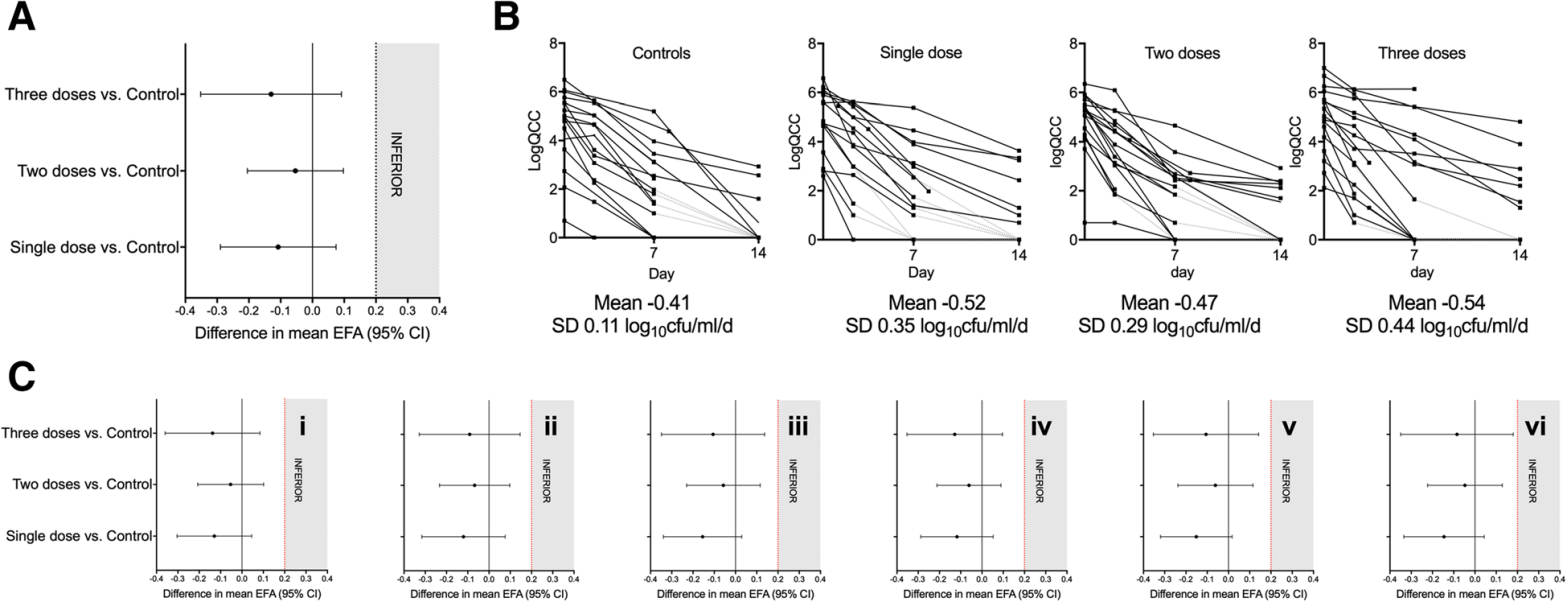
Primary and key secondary outcomes of the AMBITION Step I phase-II randomised controlled trial. The figure shows: (**a**) all three short-course treatment arms were non-inferior to control; (**b**) EFA and the individual patient slopes over the initial 14 days of treatment; and (**c**) all three short-course treatment arms remained non-inferior to control when controlling for baseline fungal burden (QCC), baseline CD4 count, baseline mental status, QCC and CD4 count, QCC count, CD4 count, mental status and QCC, CD4 count, mental status, sex, age and ART status [26]

A randomised controlled trial comparing L-AmB 3 mg/kg/day, L-AmB 6 mg/kg/day and amphotericin B deoxycholate 0.7 mg/kg/day, all given for 14 days, showed no difference in mortality outcome between any of these regimens [18]; 3 mg/kg/day is widely used as the standard dose. However, murine models suggest dosing of 3 mg/kg/day may be sub-optimal [20]. Further evidence to support this comes from the recently completed phase II AMBITION trial which was performed with the primary objective of determining the rate of cryptococcal clearance from cerebrospinal fluid (CSF), presented as Early Fungicidal Activity (EFA), of three alternative schedules of intermittent high-dose L-AmB in comparison with 14 days of standard daily L-AmB for induction therapy for HIV-associated CM [26]. Eighty participants were recruited at sites in Botswana and Tanzania and randomised to one of four treatment arms: (1) L-AmB 10 mg/kg day 1 (single dose); (2) L-AmB 10 mg/kg day 1, L-AmB 5 mg/kg day 3 (two doses); (3) L-AmB 10 mg/kg day 1, L-AmB 5 mg/kg days 3 and 7 (three doses); or (4) the control arm, being standard 14-day L-AmB (3 mg/kg/day). All treatment arms received high-dose fluconazole (1200 mg/day) for 14 days. This phase II trial was stopped by the Data Monitoring Committee (DMC) at the pre-planned interim analysis stage of 80 patients as the primary endpoint had been reached with the recommendation that the trial proceed onto the current clinical endpoint phase III trial using single dose L-AmB. The primary analysis showed that the EFA in all three short-course high-dose arms was comparable to, or greater than, the control arm, with statistical non-inferiority between all short-course arms and control at the pre-defined non-inferiority (NI) of 0.2 log_10_ colony forming units (CFU)/mL/day difference (Fig. 1). There was no evidence for any dose response effect with additional L-AmB doses, suggesting maximal fungicidal activity was achieved with a single 10 mg/kg dose. All three high-dose short-course L-AmB regimens were well tolerated, with only one Division of AIDS (DAIDS) grade IV laboratory toxicity event occurring during induction therapy, and a total of seven grade III and no grade IV clinical adverse events (AEs) associated with high-dose L-AmB. This toxicity profile compared to 33% of patients reporting grade III or IV anaemia in a combined cohort of 368 patients treated in Africa with conventional amphotericin B for 14 days [27]. There were no safety concerns with short-course treatment and no patients receiving short-course L-AmB required additional ‘rescue’ L-AmB therapy. Overall mortality in the trial was 29% at ten weeks, comparing very favourably with recent trials of amphotericin B deoxycholate-based treatments, with no significant difference between arms [17].

However, although EFA is an extremely valuable tool to rapidly screen novel antifungal treatment regimens and is associated with mortality [28], it has not been validated as a true ‘surrogate’ marker of outcome. Large phase III trials with a mortality endpoint are critical to define the optimal treatment regimens for HIV-associated CM and are essential to influence policy.

## Method/design

### Study design

The AMBITION trial is an open label, phase III, randomised controlled non-inferiority, multi-centre trial to compare single, high-dose L-AmB treatment to seven-day amphotericin B deoxycholate-based treatment for HIV-associated CM (Additional files 1 and 2).

### Hypothesis

Short-course, high-dose L-AmB given with 14 days of high-dose fluconazole and flucytosine will be non-inferior to seven days of daily-dosed amphotericin B deoxycholate given with seven days of flucytosine, followed by seven days of high-dose fluconazole, for the treatment of HIV-associated CM with all-cause mortality as the primary efficacy endpoint.

### Objectives

The primary objective is to determine whether single, high-dose L-AmB given with 14 days of high-dose fluconazole and flucytosine is non-inferior to seven days of daily-dosed amphotericin B deoxycholate given with seven days of flucytosine, followed by seven days of high-dose fluconazole in terms of all-cause mortality in HIV-associated CM patients.

### Setting

The trial will be conducted in six large referral hospitals across five countries in sub-Saharan Africa. The sites include: Princess Marina Hospital, Gaborone, Botswana; Mitchells Plain District Hospital, Cape Town, South Africa; Parirenyatwa Central Hospital, Harare, Zimbabwe; Queen Elizabeth Central Hospital, Blantyre, Malawi; Kamuzu Central Hospital, Lilongwe, Malawi; and the Infectious Diseases Institute, Kampala and Mbarara, Uganda.

### Outcome measures

The primary outcome measure is all-cause mortality within the first ten weeks after randomisation (non-inferiority). Secondary outcome measures include: EFA derived from serial lumbar punctures (LPs) on days 1, 7 and 14; proportions of patients in each arm developing clinical and DAIDS laboratory-defined grade III/IV AEs; median % change from baseline in laboratory defined parameters; PK parameters and PK/PD associations of single high-dose L-AmB; health service costs; all-cause mortality within the first two and four weeks; all-cause mortality within the first ten weeks (superiority analysis); rates of cryptococcal relapse / IRIS within the first ten weeks; and disability at ten weeks.

### Sample size

The WHO now recommends seven days amphotericin B deoxycholate-based regimens for the treatment of CM if flucytosine is available as an adjunctive antifungal. A non-inferiority design has been chosen as the primary aim of this trial is to identify an alternative safe and easy to administer short-course L-AmB treatment regimen that can be implemented in settings where giving amphotericin B deoxycholate-based treatment is difficult or impossible. An efficacious single dose L-AmB treatment would also markedly facilitate CM therapy in settings currently using amphotericin B deoxycholate-based treatment, reducing the duration of hospitalisation and the associated risks (e.g. nosocomial sepsis) and costs. Ten-week mortality in our previous trials using amphotericin B deoxycholate-based regimens at the study sites has been in the range of 28–41% [13, 29]; it was 30% with short-course high-dose L-AmB treatments in the recent phase II study. Assuming 35% ten-week mortality in both the control and test groups and using a 10% non-inferiority margin (i.e. the upper margin of the one-sided 95% CI of the difference in ten-week mortality between the two arms does not exceed 10%) and one-sided 5% type one error, 390 participants would be required per arm to achieve 90% power. This sample size will also have 83.25% power at a one-sided α = 0.025 or two-sided α = 0.05. The 10% non-inferiority margin has been chosen to ensure that only clinically unimportant differences are deemed non-inferior and is in keeping with conventional practice. If the ten-week mortality is increased to 40% the equivalent sample size is 412 per arm. Making a conservative allowance for withdrawals and losses to follow-up of up to 8% (losses are in the range of 2–4% in similar trials [10]), or a higher than anticipated mortality rate, we plan to enrol 425 participants per arm. Thus, we will randomise a total of 850 participants. This will be the largest CM treatment trial conducted in Africa.

### Inclusion and exclusion criteria

Consecutive patients aged ≥ 18 years with a first episode of CM (confirmed by either India ink or cryptococcal antigen [CrAg] test in the CSF) will be enrolled. Participants must be HIV-infected or willing to undertake an HIV test if their status is unknown. Participants must provide written informed consent or, if unable to consent, have a next of kin who agrees to the patient participating in the study, providing written consent. Pregnant (confirmed by urinary or serum pregnancy test) or lactating women, patients with a previous serious reaction to study drugs, or patients on antifungal treatment at CM treatment doses (amphotericin B deoxycholate ≥ 0.7 mg/kg or fluconazole ≥ 800 mg/day) for > 48 h or concomitant medication that is contraindicated with the study drugs at the time of assessment will be excluded.

### Consent

Written informed consent to enter the trial and be randomised will be obtained from participants or, in the case of those lacking capacity to consent, from next of kin with legal responsibility (if appropriate and in keeping with national guidance and regulations). Consent will be obtained after explanation of the aims, methods, benefits and potential hazards of the trial, and before any trial-specific procedures are performed or any blood is taken for the trial. Once the patient’s mental status improves and they regain the capacity to consent, persons enrolled via surrogate consent will be re-consented, with care taken to ensure they understand that they are: (1) free to withdraw from the research study; and (2) if they do withdraw, this will not jeopardise their future care. Patients who withdraw will revert to the standard of care at the treatment site (usually amphotericin B deoxycholate and fluconazole daily for two weeks or fluconazole monotherapy for two weeks). It will be made unambiguously clear that the participant (or guardian) is free to refuse to participate in all or any aspect of the research trial, at any time and for any reason, without incurring any penalty or affecting their access to the standard treatment available at the recruiting site (or that of their relative). Separate consent forms will be completed for the storage and/or genetic analysis of samples as determined by local guidelines. Original signed consent forms will be kept by the investigator and documented in the electronic case report form (eCRF), a copy given to the participant or family and a copy placed in the participant’s medical notes.

### Allocation

Patients will be randomised individually using a computer-generated programme. Randomisation codes will be generated via a permuted-block randomisation method and stratified by site. Block sizes will vary at four and six. Randomisation lists will be created for each site by an independent statistician and each list will be housed on the electronic data capture system (EDC) for that particular site. The full lists will be inaccessible to trial staff. Randomised allocation for each trial participant will be provided to trial staff from the randomisation list for that site. Internally, the EDC selects against the electronic randomisation and guarantees to make the selection in the natural order of the list. Once a selection is made, the randomisation record is tagged with the participant study allocated identifier, date and time of randomisation, and other EDC system audit values (username, machine name, etc).

### Interventions

**Fig. 2:**
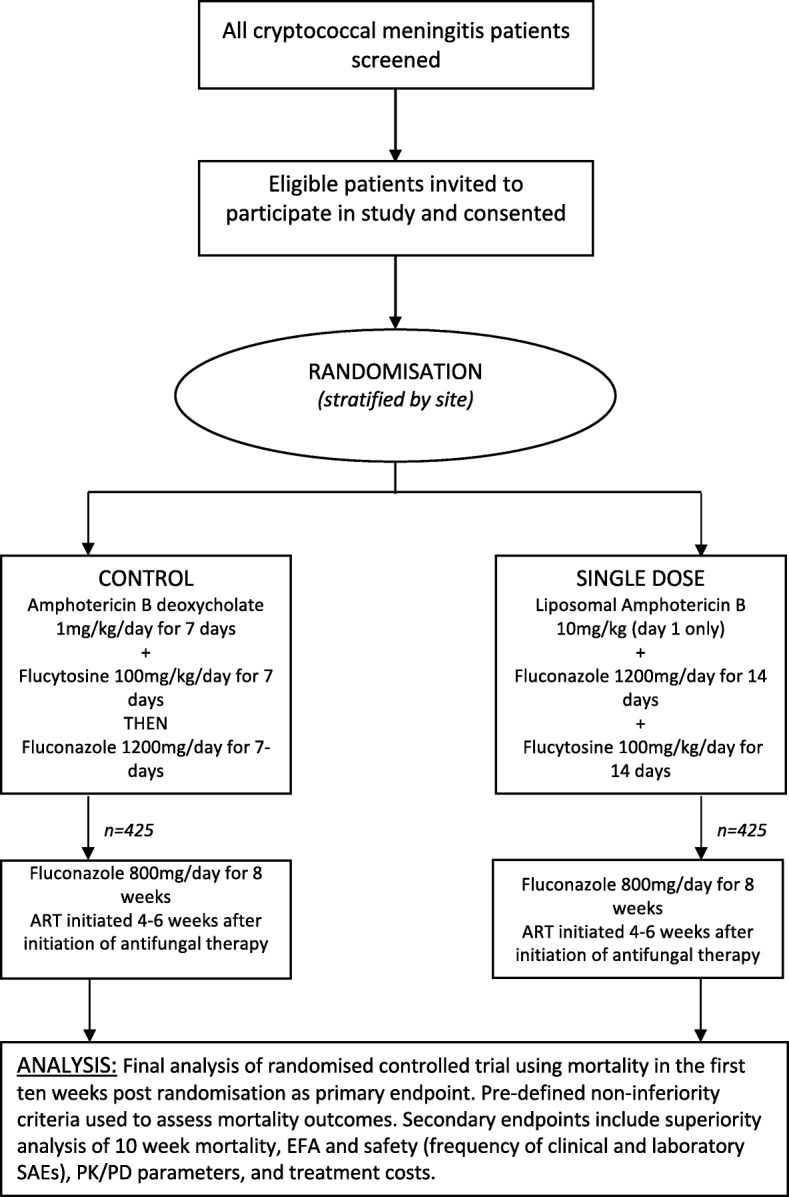
Trial schema. Trial entry, randomisation and treatment. *ART* antiretroviral therapy, *EFA* early fungicidal activity, *PD* pharmacodynamics, *PK* pharmacokinetics, *SAE* serious adverse event

Participants will be randomised to receive either intravenous L-AmB 10 mg/kg on day 1 given with 14 days of oral fluconazole 1200 mg/day and oral flucytosine 100 mg/kg/day (intervention) or intravenous amphotericin B deoxycholate 1 mg/kg/d for seven days given with seven days of oral flucytosine 100 mg/kg/day followed by seven days of oral fluconazole 1200 mg/day (control) (Fig. 2). After the two-week induction phase, all participants will then receive oral fluconazole 800 mg/day to complete ten weeks therapy and 200 mg/day thereafter. ART will be commenced four to six weeks after initiation of antifungal therapy, in line with national guidelines. Given the combination of oral and intravenous therapies, the differing duration in days of intravenous therapy and the known drug-induced toxicities that require monitoring and managing, blinding of treatment allocation was deemed to be impractical. To counter this, an objective endpoint of all-cause mortality has been chosen. In addition, all staff performing quantitative cell cultures are blind to treatment, as are coordinating investigators, including the Trial Management Group (TMG) members.

### Rescue medication

Although the results from our phase II trial demonstrate that it is unlikely that CSF fungal burden will increase after initiation of treatment, if the day 7 LP identifies an increase in CFU from baseline this will be reported as a serious adverse event (SAE) and experienced, senior clinicians at the coordinating centre will be responsible for managing this situation on a case-by-case basis to ensure all participants receive effective induction therapy.

### Schedule

All participants will be admitted to hospital for a minimum of one week. As the induction phase occurs over two weeks if participants are well enough to be discharged after day 7 and before day 14, treatment will be given under close outpatient supervision during the second week, ensuring compliance to the trial intervention and facilitating close clinical and laboratory monitoring. After the intensive phase, participants will be seen in clinic at four, six, eight and ten weeks and a single telephone follow-up to ascertain vital status and level of disability will be made at week 16. Every effort will be made (e.g. with mobile telephone calls, home visits and financial help with travelling expenses) to obtain accurate and complete follow-up data for ten weeks after the start of treatment. Particular attention will be paid to the possibility, in ART-naïve participants, of developing IRIS after starting ART [30].

Participants will have a full history and examination at baseline (Table 1). Blood will be drawn for full blood count (FBC), urea, creatinine, electrolytes and alanine transaminase (ALT). If unknown, HIV serology will be performed in addition to CD4 and viral load samples, as clinically indicated. Women of reproductive age will have a pregnancy test (urine or serum). All participants will have an LP for opening pressure, total and differential white cell count, protein, glucose, India ink, CrAg, routine culture, quantitative fungal culture and immune parameters. Further blood samples will be collected on days 3, 5, 7, 10, 12, 14 and 28 for urea and creatinine. FBC and ALT will be repeated on days 7, 14 and 28. Additional samples will be taken alongside monitoring blood tests for sub-studies, including PK/PD studies. LPs will be repeated on days 7 and 14 for opening pressure, quantitative fungal culture, CSF drug levels and immune parameters. Raised intracranial pressure will be managed with LPs as per a standard operating procedure. Cryptococcal clearance rates will be calculated using summary statistics for each patient: the rate of decrease in log_10_ CFU per mL CSF per day derived from the slope of the linear regression of log_10_ CFU against time for each patient. A linear regression model will be used to compare mean rates of decline EFA for each arm, giving summary differences with 95% CI and significance levels [31, 32]. We will adjust analyses for potential confounding factors, including baseline fungal burden. Disability at ten weeks will be assessed using two simple questions and a modified Rankin scale.

**Table 1:** Schedule of enrolment, interventions and assessments adapted from the Standard Protocol Items: Recommendations for Interventional Trials (SPIRIT) figure

	Screening	Week 1							Week 2							Week 4	Week 6	Week 8	Week 10	Week 16
Study day	≤D0	D1	D2	D3	D4	D5	D6	D7	D8	D9	D10	D11	D12	D13	D14					
Consent and randomisation																				
Eligibility criteria	X	X																		
PIS and signed consent	X	X																		
Randomisation	X	X																		
Treatment																				
Intervention: Ambisome 10 mg/kg		X																		
Intervention: Fluconazole 1200 mg/day		X	X	X	X	X	X	X	X	X	X	X	X	X	X					
Intervention: Flucytosine 100 mg/kg/day		X	X	X	X	X	X	X	X	X	X	X	X	X	X					
Control: Amphotericin B deoxycholate 1 mg/kg		X	X	X	X	X	X	X												
Control: Flucytosine 100 mg/kg/day		X	X	X	X	X	X	X												
Control: Fluconazole 1200 mg/day									X	X	X	X	X	X	X					
All participants: Fluconazole 800 mg/day															X	X	X	X	X	
All participants: 200 mg/day																			X	X
Clinical, safety and compliance assessment																				
Inpatient clinical review		X	X	X	X	X	X	X	X	X	X	X	X	X	X					
Outpatient follow-up																X	X	X	X	
Week 16 telephone																				X
Clinical labs																				
HIV testing		X																		
Pregnancy test (urine/serum)^a^		X																		
Full blood count		X						X							X	X				
CD4 count		X																		
ALT		X						X							X	X				
Urea, creatinine and electrolytes		X		X		X		X			X		X		X	X				
Drug levels		X						X												
Quantitative PCR sub-study		X		X				X							X					
Semi-quantitative cryptococcal antigen		X																		
CSF																				
Opening pressure		X						X							X					
Cell count and differential^b^		X																		
Protein, glucose^b^		X																		
Routine culture^b^		X																		
India ink examination^b,c^		X																		
Cryptococcal antigen^b,c^		X																		
Quantitative fungal culture		X						X							X					
CSF drug levels		X						X							X					
Immune parameters		X						X							X					
Semi-quantitative cryptococcal antigen		X																		

^a^For women of childbearing age; ^b^Part of routine care; ^c^India ink or cryptococcal antigen required for inclusion

### Statistical methods

The primary endpoint (all-cause mortality at ten weeks) will be analysed using a generalised linear model (GLM). The model will have treatment group as the sole predictor, a binomial distribution and an identity-link function, from which the (unadjusted) risk difference between the treatment groups and its one-sided 95% CI will be estimated. If the upper limit of the one-sided 95% CI falls below the non-inferiority margin of 10%, non-inferiority will be declared. Sensitivity analyses of the primary endpoint making different assumptions for the losses to follow-up will be conducted. Covariate-adjusted analyses for the primary endpoint will be conducted by adding pre-specified covariates into the GLM model to derive the adjusted risk difference and the upper limit of one-sided 95% CI. Imputation for baseline missing covariates will be made for the covariate-adjusted analysis. Subgroup analysis of the primary endpoint will also be performed on prespecified covariates.

The analyses of the secondary endpoints will be based on superiority test using a 5% two-sided significance level. Analyses of survival data will be conducted using unadjusted Cox regression analysis to calculate the HR and 95% CI between the treatment groups. Kaplan–Meier survival curves by treatment group will be calculated and displayed. A log-rank test will be conducted to compare the survival curves between the treatment groups. Analyses of binary secondary outcomes will be performed in a similar way as the primary endpoint analysis using GLMs with treatment group as the sole predictor. The point estimate of the treatment effect with two-sided 95% CI will be derived. The safety analysis will be descriptive and the frequency and proportions of participants suffering clinical and laboratory-defined side effects will be generated by treatment arms. Other statistical analyses may be performed if deemed necessary.

Data will be analysed using SAS 9.4 and Stata 13. Findings will be reported according to the Consolidated Standards of Reporting Trials (CONSORT) guidelines for randomised controlled trials. Primary analyses will be based on the intention-to-treat population and secondary analyses will be based on the per-protocol population. All analyses will be described in detail in the finalised and signed statistical analysis plan before data are locked and unblinding occurs.

### Dissemination of results

The results of the trial will be analysed, presented and published as soon as possible. The TMG will form the basis of the Writing Committee and will advise on the nature of the publication. The names of all investigators will be included in the authorship of any publication. An authorship policy will be agreed by all investigators before the commencement of the trial. The independent members of the Trial Steering Committee (TSC) and DMC will be listed with their affiliations in the acknowledgements or appendix sections of the main publication. The funders will have no role in the decision to publish or the content of the publication.

### Ethical approval

The Research Ethics Committee of the London School of Hygiene and Tropical Medicine have approved the protocol v2.1 07.11.17 (ref. 14,355). Approval has also been granted by the following: University of Botswana Office of Research and Development (UBR/RES/IRB/BIO/042); Botswana Ministry of Health and Wellness Health Research and Development Division (HPDME:13/18/1); Princess Marina Hospital Research and Ethics Committee (PMH 5/79(407-1-2017); University of Cape Town Human Research Ethics Committee (642/2017); Malawi National Health Sciences Research Committee (1907); Mulago Hospital Research and Ethics Committee (MHREC 1297); and the Medical Research Council of Zimbabwe (MRCZ/A/2263). Any amendments will be submitted and approved by each ethics committee.

### Timeline

In total, 850 participants will be recruited over a three-year period with a planned trial completion date of 31 December 2020. This is feasible based upon previous experience and rates of CM at the hospital sites.

### Ancillary studies

#### PK/PD

The PK/PD of L-AmB, fluconazole and flucytosine and the impact of PK variability on outcome will be described. Plasma samples will be collected at the end of the L-AmB infusion and then at 2, 4, 8, 12 and 24 h in a sub-study of participants at the Blantyre study site. A portion (0.5 mL) of the CSF sample obtained for quantitative counts will be reserved to measure fluconazole and flucytosine concentrations and thereby estimate the extent of penetration of these drugs into the CSF. Amphotericin levels will not be measured in CSF since they are known to be negligible. A PK-PD model will be constructed to explore the persistence of amphotericin B within the central nervous system and the resultant antifungal effect. Amphotericin penetration into the CNS will be estimated using compartmental modelling techniques. Monte Carlo simulation will enable further insights into the regimen(s) that may be associated with maximal antifungal activity.

#### Economic analysis

An economic analysis will be conducted to provide evidence for the cost-effectiveness of short-course L-AmB treatment. The objective of the economic analysis is to estimate the cost consequences and the cost-effectiveness of short-course L-AmB treatment compared to current care. Both societal and healthcare perspectives are chosen and health service patient costs including household costs, treatment cost and hospitalisations in both arms will be compared over the trial period in a probabilistic approach, using Monte Carlo bootstrapping methods in STATA, @Risk software and TreeAge. In the country-specific cost-consequence analyses, the societal and health service costs will be compared and used along with the trial-wide primary endpoint data to perform cost-effectiveness modelling using a decision-tree model for each country with historical data as comparison.

#### Semi-quantitative CrAg testing and diagnostic quantitative polymerase chain reaction (PCR)

A newly developed point of care, lateral flow, semi-quantitative CrAg test is now available from Institut Pasteur and Biosynex. We will use this semi-quantitative test in real time to determine antigen titre at baseline, in blood and CSF, and compare results to the currently established point of care test. Secondary trial analyses will include the association of baseline titre with outcome and exploration of the possibility of a differential treatment response between arms according to baseline titre. If such a differential response was observed, this sub-study could provide the rationale for and demonstrate the means for individualised treatment, based on a rapid assessment of antigen load. A novel diagnostic quantitative PCR (DNA and RNA) tool will be also be used in each treatment arm and correlated with quantitative culture counts. We aim to estimate the fungal load and fungal viability in blood and CSF at baseline using the PCR in addition to fungal load kinetics on treatment. The objective will be to develop a practical alternative to time-consuming quantitative cultures in order to improve detection of fungaemia and measurement of fungal burden and develop a novel biomarker for assessing the best fungicidal treatments in this and subsequent research studies.

### Quality control and assurance

Trial oversight will be provided by the TMG, TSC and Independent DMC. The study sponsor is the London School of Hygiene and Tropical Medicine. The sites will be monitored at regular intervals with visits by the trial manager/monitor in order to monitor the conduct of the trial and ensure that the principles of International Conference of Harmonisation (ICH) Good Clinical Practice (GCP) are being adhered to. Sites will be visited by an internal monitor for initiation visits before starting recruitment, after the first 10–15 participants, at 40% and 70% of recruitment targets and at trial closure, with additional visits made if required. Visits will ensure that all training has been completed, that drug supply and equipment are in place and that all staff are up to date on the protocol and procedures. A monitor from the Sponsor will visit at least three of the six sites. Central monitoring will be performed in addition to the on-site monitoring procedures. Bimonthly reports on the progress of the trial as well as the frequency of DAIDS laboratory-defined grade III/IV AEs/SAEs/suspected unexpected serious adverse events (SUSARs) will be compiled by the trial manager/statistician and reviewed by the Sponsor. All Grade IV AEs, all SAEs and all SUSARs will be reported to the TMG within 24 h [27].

### Data collection and data management

eCRF data collected and validated using the EDC will be stored in an electronic database that is protected using a scheme of authentication and encryption. Paper documents, such as clinical notes and administrative documentation, will be kept in a secure location and held for at least five years after the end of the trial. During this period, all data should be accessible to the competent or equivalent authorities, the sponsor and other relevant parties with suitable notice. Security of electronic records and data is a significant concern. All components of the distributed data systems will use authentication and encryption to render subject identity and personal health information unusable, unreadable or indecipherable to unauthorised individuals. Full drive encryption will be implemented at the hardware layer of all devices storing protected health information. A three-factor scheme will be used to authenticate users through the hardware layer to the application layer where personal health information is available. The applications will have user profiles to control access to certain data and reports. The application and database layers will use a combination of hashing and encryption for sensitive and personal data. Mobile devices and the staff operating them will not be equipped with the encryption keys to decrypt selected sensitive data fields.

### Confidentiality

We will follow the principles of the UK Data Protection Act (DPA) regardless of the countries where the trial is being conducted. Consent forms will be stored under the supervision of each local primary investigator (PI) in a secured office and accessible to trial staff only. Participants’ personal details are stored in an encrypted, separate server to the main database and participants are identified by their study number throughout the trial.

### Termination of the study

The trial will be considered closed when the last patient has completed ten weeks of active follow-up in the study, the 16-week telephonic follow-up call, and all follow-up and laboratory reports, including repeat plasma HIV viral load testing in ART failure cases, have been received. Early termination could occur if the DMC decides there is an unacceptable level of AEs in either test arm or if the intervention arm is shown to be inferior with stringent *p* value testing.

### Indemnity

The sponsor of the trial is the London School of Hygiene and Tropical Medicine and as such provides indemnity for the trial. All personnel involved in the trial will be expected to be indemnified by their employing authority. Local insurance will be taken out where local regulations require this.

## Discussion

The potential impact of a safe, sustainable regimen of high-dose L-AmB with non-inferior efficacy when compared to one week of daily-dosed amphotericin B deoxycholate would be to reduce the number of AEs seen in patients treated with amphotericin and shorten the length of hospital admissions. It is hoped that our economic analysis will demonstrate the cost-effectiveness of this intervention across all our sites in southern Africa and provide a highly favourable alternative to the current WHO-recommended first-line treatment.

### Trial status

The study is jointly funded through the European and Developing Countries Clinical Trials Partnership (EDCTP), Swedish International Development Cooperation Agency (SIDA) and Wellcome Trust / Medical Research Council (UK) / UKAID Joint Global Health Trials. Recruitment commenced in Botswana in January 2018 and in South Africa in July 2018; recruitment will commence at the other sites pending the requisite ethical and regulatory approvals.

## Additional files

Additional file 1: AMBITION Study Protocol v2.1 date: 7th November 2017. (PDF 1954 kb)

Additional file 2: AMBITION Study SPIRIT Checklist. (DOC 121 kb)

## Abbreviations

5FC: Flucytosine
ACTA: Advancing Cryptococcal Meningitis Treatment for Africa
AE: Adverse event
ALT: Alanine transaminase
AMBITION: AMBIsome Therapy Induction OptimisatioN
ART: Antiretroviral therapy
CFU: Colony-forming units
CI: Confidence interval
CM: Cryptococcal meningitis
CrAg: Cryptococcal antigen
CSF: Cerebrospinal fluid
DAIDS: Division of AIDS
DMC: Data Monitoring Committee
DPA: Data Protection Act
eCRF: Electronic case record form
EDC: Electronic data capture
EDCTP: European and Developing Countries Clinical Trials Partnership
EFA: Early fungicidal activity
FBC: Full blood count
GCP: Good Clinical Practice
GLM: Generalised linear model
HR: Hazard ratio
ICH: International Conference of Harmonisation
IRIS: Immune reconstitution inflammatory syndrome
L-AmB: Liposomal amphotericin B deoxycholate
LP: Lumbar puncture
LSHTM: London School of Hygiene and Tropical Medicine
NI: Non-inferiority
PD: Pharmacodynamics
PI: Primary investigator
PK: Pharmacokinetics
SAE: Serious adverse event
SAR: Serious adverse reaction
SUSAR: Suspected unexpected serious adverse reaction
TMG: Trial Management Group
TSC: Trial Steering Committee
USD: United States Dollar
WHO: World Health Organization
