# Correction to: AMBIsome Therapy Induction OptimisatioN (AMBITION): High Dose AmBisome for Cryptococcal Meningitis Induction Therapy in sub-Saharan Africa: Study Protocol for a Phase 3 Randomised Controlled Non-Inferiority Trial

**DOI:** 10.1186/s13063-018-3155-9

**Published:** 2019-01-14

**Authors:** David S. Lawrence, Nabila Youssouf, Síle F. Molloy, Alexandre Alanio, Melanie Alufandika, David R. Boulware, Timothée Boyer-Chammard, Tao Chen, Francoise Dromer, Admire Hlupeni, William Hope, Mina C. Hosseinipour, Cecilia Kanyama, Oliver Lortholary, Angela Loyse, David B. Meya, Mosepele Mosepele, Conrad Muzoora, Henry C. Mwandumba, Chiratidzo E. Ndhlovu, Louis Niessen, Charlotte Schutz, Katharine E. Stott, Duolao Wang, David G. Lalloo, Graeme Meintjes, Shabbar Jaffar, Thomas S. Harrison, Joseph N. Jarvis

**Affiliations:** 1 Department of Clinical Research, Faculty of Infectious and Tropical Diseases, London School of Hygiene and Tropical Medicine, London, UK; 2 Botswana-Harvard AIDS Institute Partnership, Gaborone, Botswana; 3 Research Centre for Infection and Immunity, St George’s University of London, London, UK; 4 Molecular Mycology Unit and National Reference Centre for Invasive Mycoses, Institut Pasteur, Paris, France; 5 Malawi-Liverpool-Wellcome Trust Clinical Research Programme, University of Malawi College of Medicine, Blantyre, Malawi; 6 Infectious Diseases Institute, College of Health Sciences, Makerere University, Kampala, Uganda; 7 Department of Medicine, University of Minnesota, Minneapolis, MN USA; 8 Department of Clinical Sciences and International Public Health, Liverpool School of Tropical Medicine, Liverpool, UK; 9 Department of Medicine, University of Zimbabwe College of Health Sciences, Parirenyatwa Hospital, Harare, Zimbabwe; 10 Department of Molecular and Clinical Pharmacology, University of Liverpool, Liverpool, UK; 11 Lilongwe Medical Relief Trust (UNC Project), Lilongwe, Malawi; 12 Department of Internal Medicine, University of Botswana, Gaborone, Botswana; 13 Wellcome Centre for Infectious Diseases Research in Africa (CIDRI-Africa), Institute of Infectious Disease and Molecular Medicine, and Department of Medicine, University of Cape Town, Cape Town, South Africa

**Correction to: Trials (2018) 19:649**

**https://doi.org/10.1186/s13063-018-3026-4**

Following publication of the original article [1], we have been notified that one of the author names was listed incorrectly. Both incorrect and correct author names are presented below. The original publication has been corrected.

- Originally published name:
  Sile L F Molloy
- Correct name:
  Sile F Molloy
