# Supplementary material for: AMBIsome Therapy Induction OptimisatioN (AMBITION): High Dose AmBisome for Cryptococcal Meningitis Induction Therapy in sub-Saharan Africa: Study Protocol for a Phase 3 Randomised Controlled Non-Inferiority Trial
Source: Trials. 2018 Nov 23;19:649. doi: 10.1186/s13063-018-3026-4 (PMC6251219; doi:10.1186/s13063-018-3026-4)
Supplement: Supplementary file 1 — AMBITION Study Protocol v2.1 date: 7th November 2017. (PDF 1954 kb) [file 13063_2018_3026_MOESM1_ESM.pdf]

AMBITION-cm

AMBIsome Therapy Induction OptimizatioN

High Dose AMBISOME on a Fluconazole Backbone for Cryptococcal Meningitis Induction Therapy in sub-Saharan Africa: A Phase 3 Randomised Controlled Non-Inferiority Trial

Version: **2.1**

Date: **7th November 2017**

ISRCTN #: **72509687**

Authorised by:

Name: Dr Joe Jarvis

Role: International CI

Signature: J. N. J

Date: 7th November 2017

Name: Professor Tom Harrison

Role: International CI

Signature: T Harrison

Date: 7th November 2017

GENERAL INFORMATION

This document describes the AMBITION-*cm* trial and provides information about procedures for entering participants into it. The protocol should not be used as an aide-memoire or guide for the treatment of other patients. Every care has been taken in drafting this protocol, but corrections or amendments may be necessary. These will be circulated to the registered investigators in the trial, but sites entering patients for the first time are advised to contact the co-ordinating centre to confirm they have the most up-to-date version.

COMPLIANCE

The trial will be conducted in compliance with the approved protocol, the Declaration of Helsinki 2008, the principles of Good Clinical Practice (GCP), and applicable national regulations.

SPONSOR

London School of Hygiene and Tropical Medicine (LSHTM)
Sponsor Representative: Mrs Patricia Henley, Quality & Governance Manager
Research Governance & Integrity Office

FUNDING

European & Developing Countries Clinical Trials Partnership (EDCTP)
Wellcome Trust / Medical Research Council (UK) / Department for International Development Joint Global Health Trials (JGHT)

TRIAL REGISTRATION

This trial has been registered with the ISRCTN Clinical Trials Register, where it is identified as ISRCTN#72509687.

RANDOMISATIONS
To randomise:
Upon confirmation of eligibility criteria and completion and entry of the informed consent details, the electronic data capture system (EDC) will select the next available randomization slot and present the details of the selection to the Study Doctor / Nurse on screen.
Tel: +267 390 2671 ext. 2210; 2230; 2203 or email ambition-support@bhp.org.bw for assistance. Note: Randomisation is stratified by site ONLY. The first digit of the randomisation code is the site prefix.

SAE REPORTING
Within 24 hours of becoming aware of an SAE, please email a completed SAE form to the coordinating centre on:
ambitionReporting@lshtm.ac.uk

TRIAL ADMINISTRATION

Please direct all queries to the trial manager, Nabila Youssouf, at ambition@lshtm.ac.uk in the first instance; clinical queries will be passed to the local or international PIs via the trial manager.

COORDINATING SITES

Department of Clinical Research, Faculty of Infectious and Tropical Diseases, London School of Hygiene and Tropical Medicine<br><br>Keppel Street, London, WC1E 7HT	Switchboard:<br><br>Fax:<br><br>Email:	+44 (0) 20 7636 8636<br><br>+44 (0) 20 7927 2739<br><br>ambition@lshtm.ac.uk
Cryptococcal Meningitis Group, Research Centre for Global Health, Institute of Infection and Immunity, St. George's University of London<br><br>Jenner Wing, Cranmer Terrace, London, SW17 0RE	Síle Molloy:<br><br>Fax:<br><br>Email:	+44 (0) 20 8725 5613<br><br>+44 (0) 20 8725 3487<br><br>smolloy@sgul.ac.uk
Department of Clinical Sciences and International Public Health, Liverpool School of Tropical Medicine<br><br>Pembroke Place, L3 5QA, Liverpool	Switchboard:<br><br>Fax:<br><br>Email:	+44 (0) 151 705 3100<br><br>+44 (0) 151 705 3370<br><br>shabbar.jaffar@lstmed.ac.uk

COORDINATING SITE STAFF

Clinical Lead:	David Lawrence	Tel:<br>Email:	+267 72464834<br>David.s.lawrence@lshtm.ac.uk
Clinical Advisor:	Timothée Boyer Chammard	Tel:<br>Email:	+33 616415007<br>timothee.boyer-chammard@pasteur.fr
Trial Manager:	Nabila Youssouf	Tel:<br>Email:	+44 7800 535028<br>Nabila.youssouf@lshtm.ac.uk
Project Manager:	Philippa Griffin	Tel:<br>Email:	+44 (0) 207 612 7850<br>Philippa.griffin@lshtm.ac.uk
Administrator:	[TBC]	Tel:<br>Email:	[TBC]<br>[TBC]
Database Lead:	Erik van Widenfelt	Tel:<br>Skype:<br>Email:	+267 723 31115 (primary)<br>+1 (512) 366 0233<br>erikvw3702<br>ew2789@gmail.com
Statisticians:	Duolao Wang	Tel:<br>Email:	+44 (0) 7591 521528<br>duolao.wang@lstmed.ac.uk
Epidemiologist:	Síle Molloy	Tel:<br>Email:	+44 (0) 20 8725 5613<br>smolloy@sgul.ac.uk

CHIEF INVESTIGATORS

**Dr Joseph N Jarvis**<br>Department of Clinical Research, London School of Hygiene and Tropical Medicine<br>Keppel Street, London, WC1E 7HT, UK	Tel:<br>Fax:<br>Email:	+267 765 24122<br>+44 (0) 20 7927 2739<br>joseph.jarvis@lshtm.ac.uk
**Professor Thomas S Harrison**<br>Institute of Infection and Immunity, St. George's University of London<br>Jenner Wing, Cranmer Terrace, London, SW17 0RE, UK	Tel:<br>Fax:<br>Email:	+44 (0) 20 8725 0447<br>+44 (0) 20 8725 3487<br>tharriso@sgul.ac.uk

CO-INVESTIGATORS

Professor Shabbar Jaffar	Department of International Public Health, Liverpool School of Tropical Medicine<br>Tel: +44 (0)151 705 2591<br>Email: shabbar.jaffar@lstmed.ac.uk
Professor David Lalloo	Department of Clinical Sciences and International Public Health, Liverpool School of Tropical Medicine<br>Tel: +44 (0)151 705 3179<br>Email: david.lalloo@lstmed.ac.uk
Professor Louis Niessen	Department of Clinical Sciences, Health Economics, Liverpool School of Tropical Medicine<br>Tel: +44 (0)151 705 3779<br>Email: louis.niessen@lstmed.ac.uk
Professor Duolao Wang	Department of Clinical Sciences, Biostatistics, Liverpool School of Tropical Medicine<br>Tel: +44 (0)151 705 3301<br>Email: duolao.wang@lstmed.ac.uk
Professor William Hope	Department of Molecular and Clinical Pharmacology, Institute of Translational Medicine, University of Liverpool. Tel: +44 (0)151 794 5941<br>Email: william.hope@liverpool.ac.uk
Dr Angela Loyse	Institute of Infection and Immunity, St. George's University of London<br>Tel: +44 (0)20 8725 5828<br>Email: aloyse@sgul.ac.uk
Professor Olivier Lortholary	Molecular mycology unit & National Reference Centre for Invasive Mycoses & Antifungals, Institut Pasteur<br>Tel: +33672007882<br>Email: olivier.lortholary@wanadoo.fr
Professor Francoise Dromer	Molecular Mycology Unit & National Reference Centre for Invasive Mycoses & Antifungals, Institut Pasteur<br>Tel: +33140613389<br>Email: francoise.dromer@pasteur.fr
Dr Alexandre Alanio	Molecular Mycology Unit & National Reference Centre for Invasive Mycoses & Antifungals, Institut Pasteur<br>Tel: +1 (215) 470 1130<br>Email: alexandre.alanio@gmail.com
Dr Joseph Makhema	Botswana-Harvard AIDS Institute Partnership<br>P/Bag BO 320 Gaborone, Botswana<br>Tel: +267 7210 0846, Email: jmakhema@yahoo.com

Dr Mosepele Mosepele	Botswana-Harvard AIDS Institute Partnership<br>P/Bag BO 320 Gaborone, Botswana<br>Tel: +267 7189 7691, Email: mosepele.mosepele@gmail.com
Dr Tshepo Leeme	Botswana-Harvard AIDS Institute Partnership<br>P/Bag BO 320 Gaborone, Botswana<br>Tel: +267 72985111, Email: tbleeme@yahoo.com
Professor Graeme Meintjes	Institute of Infectious Disease and Molecular Medicine and Department of Medicine, University of Cape Town, South Africa. Tel: +27 21 406 6075, Fax: +27 21 406 6796, Email: graemein@mweb.co.za
Dr Tom Crede	Department of Medicine, Mitchells Plain Hospital, AZ Berman Drive, Lentegeur, Mitchells Plain, Cape Town, South Africa. Tel: +27 21 370 4391, Email: Thomas.crede@westerncape.gov.za
Dr Charlotte Schutz	Wellcome Centre for Infectious Diseases Research in Africa, Institute of Infectious Diseases and Molecular Medicine, University of Cape Town, Faculty of Health Sciences, Anzio Road, Observatory, 7925, Cape Town, South Africa. Tel: +27 84 877 3368, Email: charlotte.schutz@uct.ac.za
Dr David Meya	Infectious Disease Institute, College of Health Sciences, Makerere University.<br>Po Box 22418 Kampala, Uganda<br>Tel: +256 772 543 730<br>Email: david.meya@gmail.com
Dr Conrad Murooza	Mbarara University of Science and Technology<br>Faculty of Medicine<br>P.O BOX 1410,<br>Mbarara, Uganda
Dr David Boulware	Infectious Disease Institute, College of Health Sciences, Makerere University.<br>Po Box 22418 Kampala, Uganda<br>Tel: +1 612 298 7963<br>Email: boulw001@umn.edu
Dr Cecilia Kanyama	Lilongwe Medical Relief Trust (UNC Project), Malawi<br>Private Bag A-104, Tidziwe Centre, Lilongwe, Malawi<br>Tel: +265 888 318 727<br>Email: ckanyama@gmail.com
Professor Mina Hosseinipour	Lilongwe Medical Relief Trust (UNC Project), Malawi<br>Private Bag A-104, Tidziwe Centre, Lilongwe, Malawi

	Tel: +265 888 202 153<br>Email: mina_hosseinipour@med.unc.edu
Dr Henry Mwandumba	MLW Programme, Queen Elizabeth Central Hospital<br>College of Medicine<br>P.O. Box 30096<br>Chichiri<br>Blantyre 3, Malawi<br>Email: Henry.Mwandumba@lstmed.ac.uk
Professor Chiratidzo E Ndhlovu	Department of Medicine<br>University of Zimbabwe College of Health Sciences<br>Parirenyatwa Hospital, Avondale, Harare<br>Tel: +263 4 791631<br>Fax: +263 4 251017<br>Email: mascen@mweb.co.zw
Dr Admire Hlupeni	Department of Medicine<br>University of Zimbabwe College of Health Sciences<br>Parirenyatwa Hospital, Avondale, Harare<br>Tel: +263 771 260 760<br>Email : ahlupeni@gmail.com

For full details of all trial committees, please see Section 18.1 - Appendix 1.

SUMMARY OF TRIAL

SUMMARY INFORMATION TYPE	SUMMARY DETAILS
**Acronym (Short Title)**	AMBITION-*cm*
**Long Title**	High Dose AMBISOME on a Fluconazole Backbone for Cryptococcal Meningitis Induction Therapy in sub-Saharan Africa: A Phase 3 Randomised Controlled Non-Inferiority Trial
**Version**	2.1
**Date**	7th November 2017
**ISRCTN #**	72509687
**Sponsor #**	QA800
**LSHTM REC #**	14355
**Study Design**	Randomised controlled trial
**Type of Participants to be Studied**	HIV-infected patients admitted with first episode of cryptococcal meningitis, identified by India ink staining of CSF or CrAg in CSF.
**Setting**	Princess Marina Hospital, Gaborone, Botswana<br>Mitchells Plain District Hospital, Cape Town, South Africa<br>Khayelitsha Hospital, Cape Town, South Africa<br>Parirenyatwa Central Hospital, Harare, Zimbabwe<br>Queen Elizabeth Central Hospital, Blantyre, Malawi<br>Kamuzu Central Hospital, Lilongwe, Malawi<br>Mulago Hospital, Infectious Disease Institute, Kampala, Uganda<br>Mbarara Hospital, Mbarara, Uganda
**Interventions to be Compared**	1. L-AmB 10 mg/kg day 1 (single dose) with flucytosine 100mg/kg/d and fluconazole 1200mg/day for 14-days<br>2. Amphotericin-B deoxycholate 1mg/kg/d for 7-days with flucytosine 100mg/kg/d(standard dose “control arm”)* followed by 7-days of fluconazole 1200mg/day.
**Study Hypothesis**	Single high-dose L-AmB given with 14-days of high-dose fluconazole will be non-inferior to 7-days of standard dosed Amphotericin B deoxycholate followed by 7-days of high dose fluconazole induction therapy, both given with flucytosine, for the treatment of HIV-associated cryptococcal meningitis in averting all-cause mortality.
**Primary Outcome Measure(s)**	1. All-cause mortality within the first 10 weeks after randomisation (non-inferiority)
**Secondary Outcome Measure(s)**	1. Early Fungicidal Activity<br>2. Proportions of patients developing clinical and laboratory-defined grade III/IV adverse events; median % change from baseline in laboratory defined parameters<br>3. PK parameters and PK/PD associations<br>4. Health service costs by treatment arm<br>5. All-cause mortality within the first 2 and 4 weeks<br>6. All-cause mortality within the first 10 weeks (superiority)<br>7. Rates of cryptococcal relapse / IRIS within the first 10 weeks

	8. Disability at 10 weeks
**Randomisation**	Individual randomisation using SAS PROC PLAN via a permuted-block randomisation method stratified by site. Block sizes will vary.
**Number of Participants to be Studied**	850 participants<br>(425 participants per arm)
**Duration**	4 years
**Ancillary Studies/Sub studies**	PK/PD, cost effectiveness, diagnostics, immunology, transcriptomics, host and pathogen genetics, isolate phenotyping, outcomes among ART exposed individuals.
**Sponsor**	London School of Hygiene and Tropical Medicine
**Funder**	European & Developing Countries Clinical Trials Partnership, Wellcome Trust, Medical Research Council (UK), Department for International Development (UK)
**Trial Manager**	Dr Nabila Youssouf
**Chief Investigators**	Dr J N Jarvis<br>Professor T S Harrison
**Site PI (Malawi - Lilongwe)**	Professor M Hosseinipour / Dr C Kanyama
**Site PI (Malawi - Blantyre)**	Professor David Lalloo / Dr Henry Mwandumba
**Site PI (Uganda)**	Dr D Meya / Professor D Boulware
**Site PI (Botswana)**	Dr M Mosepele / Dr T Leeme
**Site PI (South Africa)**	Professor G Meintjes
**Site PI (Zimbabwe)**	Professor C E Ndhlovu

TRIAL SCHEMA

Figure 1. Trial Entry, Randomisation and Treatment

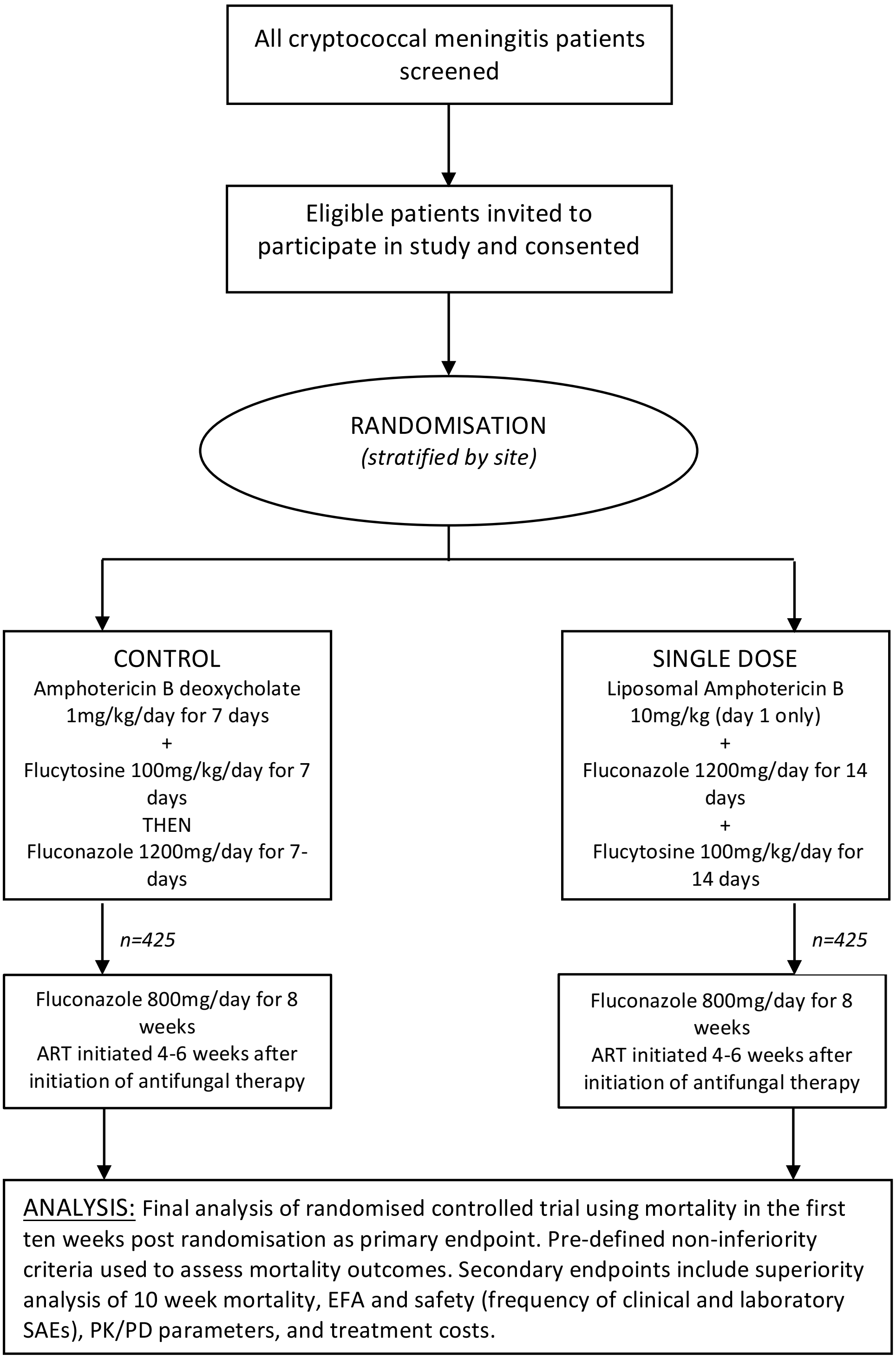

TRIAL SUMMARY

LAY SUMMARY

Cryptococcal meningitis is a leading cause of death in HIV-infected individuals in Africa. The current recommended treatment is a drug called amphotericin B deoxycholate (D-AmB). Current guidelines recommend treatment with amphotericin B deoxycholate which requires at least 7 days of intravenous infusions given in hospital, making it difficult and costly to administer. It also causes many side effects, including kidney impairment and anaemia, making close laboratory monitoring essential. The combination of the costs associated with prolonged hospital admissions, the difficulties in administration and the need for laboratory monitoring make amphotericin B deoxycholate treatment difficult in much of Africa.

A modified form of amphotericin B is available called liposomal amphotericin B (Ambisome or L-AmB). This is considerably less toxic than standard amphotericin B deoxycholate, and is known to be efficacious in treatment of cryptococcal meningitis. Its use has been limited by the high cost of therapy, but recent data suggest that much shorter courses of L-AmB may be effective in the treatment of cryptococcal meningitis. Due to its lower toxicity, higher doses of L-AmB can be given safely, and it also persists for a long time in the tissues, raising the possibility of delivering highly effective induction therapy with just a single dose. A large reduction in the number of doses and duration of hospitalisation, together with reduced pricing of L-AmB, may result in cryptococcal meningitis treatment costs that are not more than those with 1 week of conventional amphotericin B deoxycholate, and provide a convenient, safe and efficacious alternative to conventional amphotericin B therapy.

This study aims to define a new easy to administer, effective, and cost-effective schedule for L-AmB use in the treatment of cryptococcal meningitis. A preliminary small study has shown that a single high dose of L-AmB is as effective at clearing cryptococcal infection from the cerebrospinal fluid (CSF) as 14-day courses. This study will determine whether a single high dose of L-AmB is as effective as the standard treatment in terms of preventing deaths from cryptococcal meningitis.

ABSTRACT AND SUMMARY OF TRIAL DESIGN

Type of design
An open label phase III randomised controlled non-inferiority trial to compare single dose L-AmB treatment to 7-day amphotericin B deoxycholate based treatment for HIV-associated cryptococcal meningitis.

Disease/patients studied
Patients diagnosed with a first episode of cryptococcal meningitis (CM) based on cerebrospinal fluid (CSF) India ink stain or cryptococcal antigen (CrAg) testing. If not known, HIV-seropositivity to be confirmed.

Trial interventions
1. L-AmB 10 mg/kg day 1 (“single dose”) given with fluconazole 1200mg/day plus flucytosine 100mg/kg/d for 14-days.
2. Amphotericin B deoxycholate 1 mg/kg/d for 7-days given with flucytosine 100mg/kg/d (standard dose, “control arm”) followed by fluconazole 1200mg/day for 7-days.

All patients receive fluconazole 800 mg/d to 10 weeks (provision will be ensured throughout the trial), and 200 mg/d thereafter (as per national guidelines at the site). ART will be commenced 4 – 6 weeks after initiation of antifungal therapy.

OUTCOME MEASURES

Primary:
All-cause mortality within the first 10 weeks (non-inferiority)

Secondary:
1. Early Fungicidal Activity derived from serial lumbar punctures (LPs) on days 1, 7 and 14.
2. Proportions of patients in each arm developing clinical and laboratory-defined grade III/IV adverse events; median % change from baseline in laboratory defined parameters.
3. PK parameters and PK/PD associations of single high dose L-AmB.
4. Health service costs.
5. All-cause mortality within the first 2 and 4 weeks
6. All-cause mortality within the first 10 weeks (superiority)
7. Rates of cryptococcal relapse / IRIS within the first 10 weeks
8. Disability at 10 weeks

Duration
Standard induction therapy for CM is 14 days. In the control arm this will consist of 7-days of daily amphotericin B deoxycholate infusions plus oral flucytosine 100mg/kg/day followed by 7-days of high-dose (1200mg/day) oral fluconazole. In the short-course arm this will consist of a single dose of L-AmB plus 14-days of high dose (1200mg/day) oral fluconazole and oral flucytosine 100mg/kg/day. Consolidation phase treatment with fluconazole 800mg daily will continue for a further 8 weeks in all patients. Patients will be admitted and clinical responses monitored daily for the first 2 weeks or until discharge. Outpatient follow-up will be at 4, 6, 8 and 10 weeks, with a final telephone follow-up at 16 weeks.

ANCILLARY STUDIES/SUBSTUDIES

PK/PD studies and cost-effectiveness studies will be performed as outlined in this protocol. Cryptococcal isolates will be saved and shared with collaborators and members of the cryptococcal research community for ongoing phenotypic and molecular epidemiology studies. Blood and CSF samples will be saved for ongoing studies examining the phenotype of the immune response to Cryptococcus, and the pathophysiology of immune reconstitution inflammatory syndromes. Blood will also be collected from a sub-group of individuals for studies determining how polymorphisms in immune response genes relate to susceptibility to and outcomes from cryptococcal meningitis. An evaluation will be undertaken of the utility of baseline cryptococcal antigen titre assessment by semi-quantitative CrAg testing and quantitative PCR. Any samples that require shipment will be handled in accordance with the local guidelines and relevant ethical approval will be obtained. Patients who have been exposed to ART will be followed-up with repeat viral loads and resistance testing, as per the standard procedures at the sites, and data collected about rates of treatment failure and drug resistance.

CONTENTS

GENERAL INFORMATION 2

SUMMARY OF TRIAL 8

TRIAL SCHEMA 10

TRIAL SUMMARY 11

LAY SUMMARY 11
ABSTRACT AND SUMMARY OF TRIAL DESIGN 11
OUTCOME MEASURES 12
ANCILLARY STUDIES/SUBSTUDIES 12

CONTENTS 13

ABBREVIATIONS AND GLOSSARY 16

BACKGROUND 19

1.1 RATIONALE 19
1.2 POPULATION 20
1.3 OBJECTIVES 20
1.4 RELEVANT STUDIES/TRIALS 21
1.5 RISKS AND BENEFITS 25

SELECTION OF SITES/CLINICIANS 27

2.1 SITE/INVESTIGATOR INCLUSION CRITERIA 27
2.1.1 PI's Qualifications & Agreements 28
2.1.2 Adequate Resources 28
2.1.3 Site Assessment 28
2.2 SITE/INVESTIGATOR EXCLUSION CRITERIA 29
2.3 APPROVAL AND ACTIVATION 29
2.4 SITE MANAGEMENT 29

SELECTION OF PATIENTS 30

3.1 PATIENT INCLUSION CRITERIA 30
3.2 PATIENT EXCLUSION CRITERIA 30
3.3 NUMBER OF PATIENTS 30
3.4 CO-ENROLMENT GUIDELINES 30
3.5 SCREENING PROCEDURES & PRE-RANDOMISATION INVESTIGATIONS 31

REGULATION & RANDOMISATION 32

4.1 RANDOMISATION PRACTICALITIES 32
4.2 RANDOMISATION CODES & UNBLINDING 32
4.3 CO-ENROLMENT GUIDELINES 32

TREATMENT OF PATIENTS 33

5.1 BASELINE ASSESSMENT 33
5.2 ANTIFUNGAL DRUG TREATMENT 33
5.2.1 Drug Regimens 33
5.2.2 Source of drugs...34
5.2.3 Dispensing 34
5.2.4 Dose Modifications, Interruptions & Discontinuations 34
5.2.5 Accountability & Unused Drugs/Devices...34
5.2.6 Compliance & Adherence...34
5.3 OTHER TREATMENT... 35
5.3.1 Prehydration and electrolyte supplementation 35
5.3.2 Management of raised CSF opening pressure...35
5.3.3 Antiretroviral treatment..35
5.4 PROTOCOL TREATMENT DISCONTINUATION.. 35
5.5 TREATMENT DATA COLLECTION 36
5.6 NON-TRIAL TREATMENT... 36
5.6.1 Medications Permitted...36
5.6.2 Medications Not Permitted..36
5.6.3 Medications to be Used With Caution 36
5.6.4 Treatment After Trial Event..36
5.7 CO-ENROLMENT GUIDELINES.. 36

ASSESSMENTS & FOLLOW-UP 37

6.1 TRIAL ASSESSMENT SCHEDULE.. 37
6.2 PROCEDURES FOR ASSESSING EFFICACY 38
6.3 PROCEDURES FOR ASSESSING SAFETY... 38
6.4 PROCEDURES FOR ASSESSING PHARMACOKINETICS 39
6.5 OTHER ASSESSMENTS.. 40
6.6 EARLY STOPPING OF FOLLOW-UP 41
6.7 PATIENT TRANSFERS... 42
6.8 LOSS TO FOLLOW-UP 42
6.9 ASSESSMENTS AT TRIAL CLOSURE... 42

SAFETY REPORTING 43

7.1 DEFINITIONS.. 43
7.1.1 Medicinal Products...44
7.1.2 Adverse Events 44
7.1.3 exempted Adverse Events 44
7.1.4 Disease-related Events 44
7.1.5 Other Study-specific Requirements...44
7.2 OTHER NOTABLE EVENTS 44
7.2.1 Pregnancy...44
7.3 INVESTIGATOR RESPONSIBILITIES.. 45
7.3.1 Investigator Assessment...45
7.3.2 Notification Procedure 46
7.4 SPONSOR RESPONSIBILITIES 47

QUALITY ASSURANCE & CONTROL..48

8.1 RISK ASSESSMENT 48

8.2 CENTRAL MONITORING AT THE CO-ORDINATING CENTRE 48
8.3 ON-SITE MONITORING 48
8.3.1 Direct Access to Patient Records 48
8.3.2 Confidentiality 48

STATISTICAL CONSIDERATIONS 49

9.1 METHOD OF RANDOMISATION 49
9.2 OUTCOME MEASURES 49
9.3 SAMPLE SIZE 49
9.4 INTERIM MONITORING & ANALYSES 50
9.5 ANALYSIS PLAN (BRIEF) 50

ANCILLARY STUDIES 52

REGULATORY & ETHICAL ISSUES 53

11.1 COMPLIANCE 53
11.1.1 Regulatory Compliance 53
11.1.2 Site Compliance 53
11.1.3 Data Collection & Retention 53
11.2 ETHICAL CONDUCT OF THE STUDY 54
11.2.1 Ethical Considerations 54
11.2.2 Ethical Approvals 54
11.3 COMPETENT AUTHORITY APPROVALS 54
11.4 OTHER APPROVALS 54

INDEMNITY 56

FINANCE 57

OVERSIGHT & TRIAL COMMITTEES 58

14.1 TRIAL MANAGEMENT GROUP (TMG) 58
14.2 TRIAL STEERING COMMITTEE (TSC) 58
14.3 INDEPENDENT DATA MONITORING COMMITTEE (IDMC) 58

PUBLICATION 59

PROTOCOL AMENDMENTS 60

REFERENCES 65

APPENDICES 69

18.1 APPENDIX 1 - DIVISION OF AIDS TABLE FOR GRADING THE SEVERITY OF ADULT AND PEDIATRIC ADVERSE EVENTS 69

ABBREVIATIONS AND GLOSSARY

AE	Adverse event
AIDS	Acquired Immune Deficiency Syndrome
ALT	Alanine transaminase
AmB	Amphotericin B
AR	Adverse reaction
ART	Antiretroviral therapy
ARV	Antiretroviral
BHP	Botswana Harvard Partnership
CDC	Centres for Disease Control and Prevention
CF	Consent Form
CFU	Colony forming units
CI	Chief Investigator
CI	Confidence interval
CM	Cryptococcal meningitis
CrAg	Cryptococcal antigen
CSF	Cerebrospinal fluid
CTA	Clinical Trials Authorisation
D-AmB	Amphotericin B deoxycholate
DM	Data Manager
DMC	Data Management Centre
DPA	(UK) Data Protection Act
eCRF	Electronic Case Report Form
EDC	Electronic Data Capture system
EDCTP	European Developing Countries Clinical Trials Partnership
EFA	Early Fungicidal Activity
EU	European Union

FBC	Full Blood Count
FDA	(US) Food and Drug Administration
GCP	Good Clinical Practice
HIV	Human Immunodeficiency Virus
HR	Hazard ratio
IB	Investigator Brochure
ICH	International Conference on Harmonisation of Technical Requirements for Registration of Pharmaceuticals for Human Use
IDI	Infectious Diseases Institute
IDMC	Independent Data Monitoring Committee
IMP	Investigational medicinal product
IRB	Institutional Review Board
ISRCTN	International Standard Randomised Controlled Trial Number
IRIS	Immune Reconstitution inflammatory syndrome
ITT	Intention-to-treat
L-AmB	Liposomal Amphotericin B
LP	Lumbar Puncture
LSHTM	London School of Hygiene and Tropical Medicine
LSTM	Liverpool School fo Tropical Medicine
NIH	National Institutes of Health
NIMP	Non-investigational-medicinal product
OD	Once daily
PBMC	Peripheral blood mononuclear cells
PI	Principal Investigator
PIS	Patient Information Sheet
PD	Pharmacodynamics
PK	Pharmacokinetics

R&D	Research and Development
RCT	Randomised controlled trial
REC	Research Ethics Committee
SAE	Serious adverse event
SAR	Serious adverse reaction
SD	Standard deviation
SOP	Standard operating procedure
SPC	Summary of Product Characteristics
SSA	Site-specific approval
SUSAR	Suspected unexpected serious adverse reaction
TM	Trial Manager
TMF	Trial Master File
TMG	Trial Management Group
TSC	Trial Steering Committee
UAR	Unexpected adverse reaction
VL	Viral load
WHO	World Health Organization
WMA	World Medical Association

BACKGROUND

1.1 RATIONALE

Early mortality among people initiating HIV-treatment in Africa is considerably higher than in high-income countries[1-4]. CM is a major cause of mortality in HIV-programmes in Africa, associated with 10-20% of all deaths[5]. Despite ART roll-out approximately half of HIV-infected individuals in Sub-Saharan Africa are not on ART and about one-third of HIV-infected individuals still present for care with very low CD4 counts. The incidence of opportunistic co-infections such as CM in this group is high[6]. CM remains the most common cause of adult meningitis in much of Africa[7]. Furthermore, the number of CM cases are not decreasing despite ART access, and now include both ART naïve and ART experienced patients, with half of CM patients presenting having had prior exposure to ART but with persisting low CD4 counts due to non-adherence and/or ART failure[8,9]. The poor outcomes reported using currently available antifungal therapy in African centres are a critical driver of this high mortality. Mortality using amphotericin B deoxycholate-based therapy in Africa, even in clinical trial settings, remains in the region of 35-45%[10-12]. Amphotericin B deoxycholate-based therapy requires hospitalization for at least 7-14 days, and its toxicity profile requires costly laboratory monitoring. The average hospitalization cost for CM treated with amphotericin B deoxycholate is $800-1000 in Zimbabwe where the annual per capita GDP is under $1000. In most of sub-Saharan Africa, the lack of access to reliable laboratory monitoring and limited nursing capacity make safely administering conventional amphotericin B deoxycholate difficult or impossible. Consequently, amphotericin B deoxycholate-based therapy is generally not routinely available in Africa, outside of South Africa. Fluconazole, the oral alternative widely used in Africa, even at a dosage up to 1200 mg/d, is much less rapidly fungicidal than amphotericin B and mortality at 10 weeks with fluconazole treatment is around 60%[13,14]. Given that HIV-prevalence and incidence in southern and east Africa remain high, inadequate ART coverage, coupled with poor monitoring of individuals on ART resulting in treatment failure and limited access to screening and pre-emptive treatment for CM, CM will remain a major cause of morbidity and mortality in the region for the foreseeable future and new treatment strategies are urgently needed.

International treatment guidelines have previously recommended 2-week courses of amphotericin B deoxycholate-based treatment. However data from two of our recent studies using shorter-course conventional amphotericin B deoxycholate (5-7 days) demonstrate comparable rates of clearance of infection and clinical outcomes to 14-day amphotericin B deoxycholate-based regimens (see Section 1.4), with large gains in tolerability[14,15]. Animal model data also support the rationale for short-course induction therapy with amphotericin B deoxycholate. Recent mouse model work suggests that amphotericin B deoxycholate courses as short as 3 days may be as effective as 14-day courses. These murine pharmacokinetic studies suggest that amphotericin B concentrations progressively accumulate in the brain despite plasma concentrations being at "steady state" (see Section 1.4), as the physicochemical properties of amphotericin B enable it to "stick" in the lipid-rich cerebrum and not be readily released back into the circulation[16]. This theory has been furthered by the recently completed clinical-endpoint Advancing Cryptococcal Meningitis Treatment for Africa (ACTA) trial which demonstrated that 1-week of amphotericin B deoxycholate and flucytosine is superior to 2-weeks of treatment in averting all-cause mortality in patients with first episode of HIV-associated CM (HR 0.56 (0.35-0.91)[53]. As a consequence of these findings, guidelines now recommend a 1 week course of amphotericin B deoxycholate-based treatment with flucytosine.

L-AmB, a newer lipid-based formulation of amphotericin B, is particularly suited to being used as part of a short-course yet highly effective induction treatment for HIV-associated CM, due to (a) the potential for high dosing made possible by the lower rates of nephrotoxicity, and (b) the long tissue half-life. The long tissue half-life of L-AmB following high dose administration in patients is well-established[17-20], as is its effective penetration into brain tissue[21]. The concept of single or intermittent

dosing with very high doses is also established in both prophylaxis in haematology patients[19,20] and treatment of visceral leishmaniasis in lower and middle income countries[22]. Single doses of up to 15mg/kg have been safely given[19], and doses of 10mg/kg are routinely given and have been shown to be efficacious for treatment of visceral leishmaniasis[22] and invasive fungal infections[23]. Even at high doses, L-AmB is associated with significantly less nephrotoxicity and anaemia, and lower rates of infusion reactions than conventional amphotericin B deoxycholate[24] (14-day courses of conventional amphotericin B deoxycholate are associated with an average drop in haemoglobin of 2.3 g/dl, and a mean increase in creatinine of 73% in the context of HIV-associated CM[25]). Pharmacokinetic data from animal models[18] and humans[17] suggest that increasing L-AmB dosing from the currently recommended 3-4 mg/kg may lead to improved outcomes, and, as with standard amphotericin B, that intermittent dosing regimens may be as effective as daily therapy[18](figure 2). Although L-AmB is recommended as treatment for HIV-associated CM in several national guidelines[26,27], optimal dosing is unknown[24] and the strategy of short-course high dosing of L-AmB has never been tested in a phase III clinical trial.

We have recently completed a phase-II study examining the Early Fungicidal Activity (EFA) of three short-course high-dose L-AmB schedules for the treatment of HIV-associated CM in Tanzania and Botswana[28,29]. The study was designed to help us identify the optimal dose of L-AmB to evaluate in a Phase-III trial. The three test schedules were (i) L-AmB 10 mg/kg day 1 (single dose); (ii) L-AmB 10 mg/kg day 1, L-AmB 5 mg/kg day 3 (two doses); (iii) L-AmB 10 mg/kg day 1, L-AmB 5 mg/kg days 3, and 7 (three doses). The control arm was standard 14-day L-AmB (3mg/kg/d). All were given with high dose fluconazole 1200mg/day[30,31]. This phase-II study demonstrated that all short course high dose L-AmB regimens were very well tolerated, and showed rapid clearance of infection and low mortality rates in the single dose arm, hence we have selected the single dose as the short-course regimen for this phase-III trial (see Section 1.4). However, although EFA is an extremely valuable tool to rapidly screen novel antifungal treatment regimens, and is associated with mortality, it has not been validated as a true "surrogate" marker of outcome. Large phase-III trials with a mortality outcome, such as we propose here, are critical to define the optimal treatment regimens for HIV-associated CM and are essential in order to influence policy[30].

1.2 POPULATION

The study population will be HIV-seropositive patients admitted with a first episode of cryptococcal meningitis, identified by India ink staining of CSF or CrAg in CSF, at the participating centres, who fulfil the inclusion/exclusion criteria outlined in Section 3.

1.3 OBJECTIVES

Primary Objective:
To determine whether short-course high-dose L-AmB is as effective as 7-day amphotericin B deoxycholate-based treatment courses (current standard of care) in averting all-cause mortality in HIV-associated CM patients in a phase-III clinical endpoint trial.

Secondary Objectives:

1. To determine the EFA in both treatment arms.
2. To examine the proportions of patients in each arm with clinical and laboratory-defined grade III/IV adverse events; median % change from baseline in laboratory defined parameters, by treatment arm.
3. To determine PK parameters and PK/PD associations of single high dose L-AmB.
4. To determine health service costs by treatment arm.
5. To determine all-cause mortality within the first 2 and 4 weeks.
6. To determine whether short-course high-dose L-AmB is superior to 7-day amphotericin B-based

treatment courses (current standard of care) in averting all-cause mortality.

7. To determine rates of cryptococcal relapse / IRIS within the first 10 weeks by treatment arm.
8. To determine rates of disability at 10 weeks by treatment arm.

Objectives relating to the addition substudies are described in Section 6.5 Other Assessments.

Methodology:
An open-label multi-centre phase-III randomised controlled non-inferiority trial.

1.4 RELEVANT STUDIES/TRIALS

The feasibility and power of using serial quantitative CSF cultures to rapidly assess the fungicidal activity ("early fungicidal activity", or "EFA") of new antifungal combinations have been demonstrated in a series of trials performed by our group over the last decade. Over 1500 patients have now been studied using the technique which has been shown to be both practical and safe, and enables fungicidal activity to be compared in multiple drug regimens using relatively small numbers of patients prior to progression to phase 3 studies. Furthermore, analysis of aggregate data from these trials shows that the rate of clearance of infection based on quantitative cultures is also associated with clinical outcome, independently of other major prognostic indicators (Figure 1), further justifying the use of EFA as both a statistically powerful and clinically meaningful endpoint in phase II trials[30,32,33]. However, whilst EFA is an extremely valuable tool to rapidly screen novel antifungal treatment regimens, and is strongly associated with mortality, it has not yet been sufficiently validated as a true "surrogate" marker of outcome, hence large mortality endpoint studies are still required to define optimal treatment regimens for HIV-associated cryptococcal meningitis.

Until recently treatment guidelines recommended 2-week courses of amphotericin B deoxycholate based treatment, however there are data from two phase II studies, using shorter-course conventional D-AmB (5-7 days), with high dose fluconazole, demonstrating no slowing of the rate clearance of infection during the second week of treatment (after amphotericin B had been discontinued) (Figure 1). Additionally, there were large gains in tolerability and therefore sustainability[14,15,25]. The recently completed ACTA trial has now tested short 7-day course amphotericin B deoxycholate based treatment with flucytosine in a clinical endpoint trial, and shown mortality outcomes that are statistically superior compared with those achieved with 14-day courses (HR 0.56 (0.35-0.91)). These findings were presented at the International AIDS Society Conference on HIV Science, Paris, July 2017 and have subsequently been incorporated into updated World Health Organisation guidelines (in press). The ACTA study also confirmed that flucytosine (5FC) is a significantly superior partner drug for amphotericin B based treatments compared with fluconazole, leading to a substantial mortality reduction of 32% (95%CI 16 - 55%, p=0.002)[53]

There are also animal model data further supporting the rationale for short course induction therapy with amphotericin B deoxycholate. Recent data from experimental models of cryptococcal meningitis in mice and rabbits suggest that an abbreviated regimen of amphotericin B (as short as just 3 days) is as effective as 14-day courses. These experimental studies suggest that there is progressive accumulation of amphotericin B in the cerebrum, which is potentially related to the physicochemical properties of amphotericin B that enable it to "stick" in the lipid-rich cerebrum (Figure 2[16]). These persistent concentrations in the brain cause sustained antifungal activity.

Figure 1. Fall in CSF *C. neoformans* CFU over time in thirty HIV-seropositive, antiretroviral therapy-naïve, patients with first episode cryptococcal meningitis were treated with high dose fluconazole (1200 mg/d for 2 weeks, then 800 mg/d until ART started) plus amphotericin B (D-AmB, 1 mg/kg/d), with routine normal saline and potassium supplementation, for the initial 5 days. The decrease in log CFU per ml CSF per day was calculated for each patient using the slope of the linear regression of log CFU against time. Early fungicidal activity (EFA) is shown as the mean rate of fall in log CFU counts/day. The mean rate of decrease in CFU per ml CSF per day, or early fungicidal activity (EFA), was −0.30 ± 0.11 log CFU/day calculated over the first 2 weeks of treatment, and −0.31 ± 0.14 log CFU/day calculated over the first week[16].

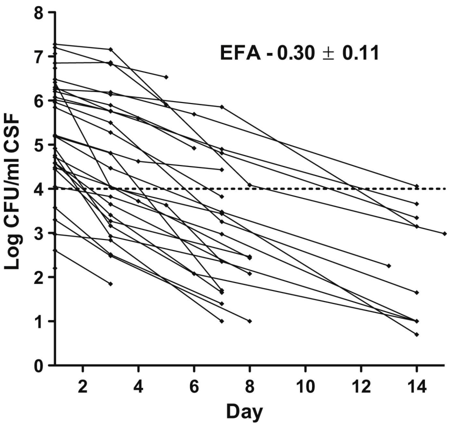

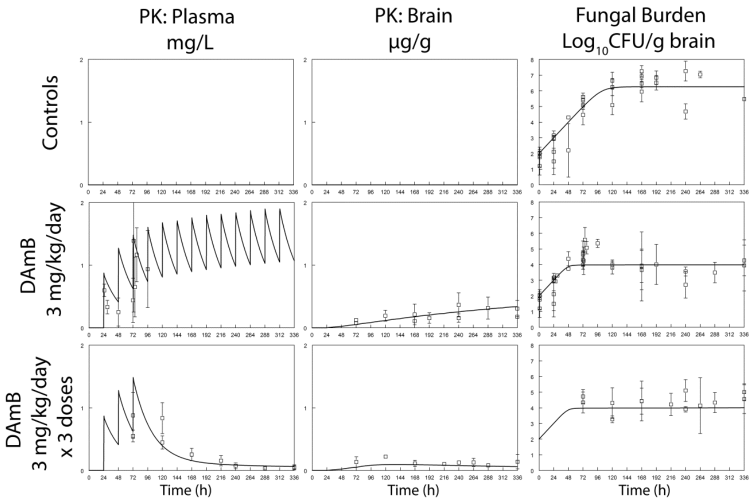

Figure 2 (a). The pharmacokinetics and pharmacodynamics of amphotericin B against *Cryptococcus neoformans* in a murine model of cryptococcal meningitis. There is hysteresis in the pharmacokinetics in plasma versus cerebrum with concetrations in the latter progressively increasing throughout the experimental period. The administration of an abbreviated regimen results in persistently detectable concentrations in both plasma and the cerebrum, which accounts for the persistent antifungal effect. Data are mean ± standard deviation. The solid line is the fit of the mathematical model to the data.

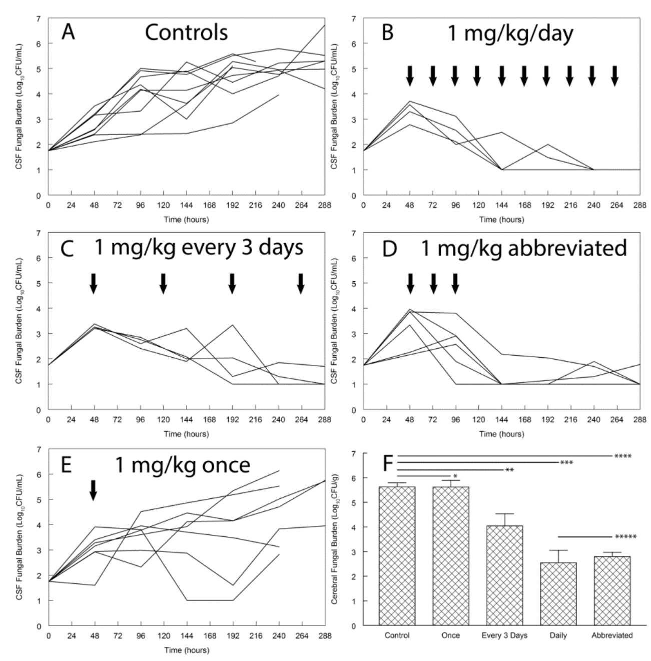

Figure 2 (b). The effect of various regimens of amphotericin B for experimental cryptococcal meningoencephalitis. Amphotericin B 1 mg/kg was administered daily (Panel B), every 3rd day (Panel C), as an abbreviated regimen for three consecutive days (Panel D) and once (Panel E). The solid arrows show the time of drug administration. The data in Panels A-E represent the time course of infection in the CSF. Each line represents the fungal burden in an individual rabbit. Panel F shows the fungal density in the cerebrum at the end of the experiment. Each bar represents the mean fungal density ± standard error of the mean. There is no statistically significant difference in the cerebral fungal density of rabbits receiving daily therapy versus those receiving an abbreviated regimen of 1 mg/kg at 48, 72 and 96 hours relative to inoculation. * p=1.00 (not significant), ** p=0.006, *** p=<0.001, ****p=<0.001, ***** p=1.00 (not significant).

L-AmB, due to the a) higher dosing made possible by the lower rates of nephrotoxicity, and b) the long tissue half-life, is particularly suited to being used as part of a short course yet highly effective induction treatment for HIV-associated cryptococcal meningitis. The long tissue half-life of L-AmB following high dose administration in patients is well established[17-20], as is its effective tissue penetration (including brain tissue)[21]. The concept of single or intermittent dosing is also established in both prophylaxis in haematology patients[19,20] and in treatment of visceral leishmaniasis[22]. Single doses as high as 15mg/kg have been safely given[19]. Doses of 10mg/kg are routinely given and have been shown to be efficacious for treatment of visceral leishmaniasis[22] and invasive fungal infections[23]. Pharmacokinetic data from both animal models[18] and humans[17] suggest that increasing L-AmB dosing from the currently recommended 3mg/kg may be required to facilitate safe, easy to administer, and effective intermittent dosing regimens and produce an antifungal effect that is as least as good as standard daily therapy (Figure 3).

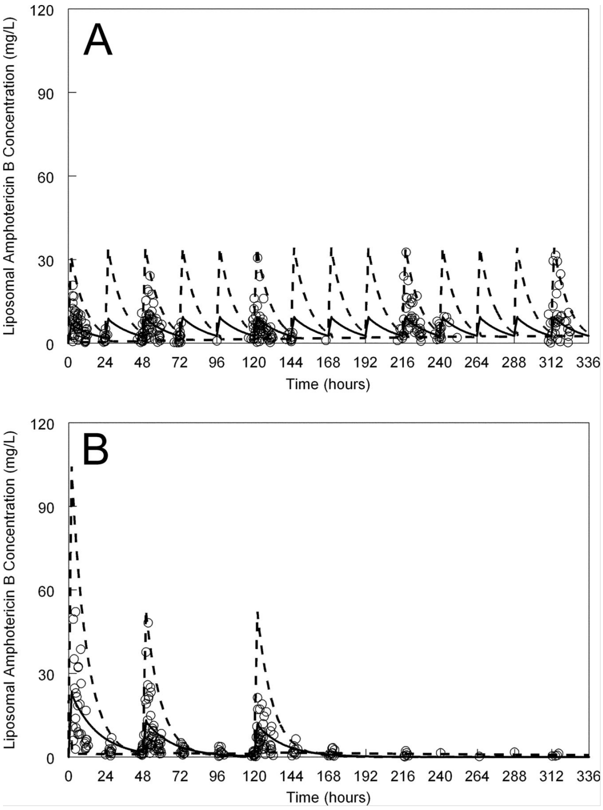

Figure 3 (a). Pharmacokinetics of liposomal amphotericin B administered at different doses. Liposomal amphotericin B was given at a dose of 3 mg/kg/day (A) and in doses of 10 mg/kg, 5 mg/kg, and 5 mg/kg at 0, 48, and 120 h, respectively (B). The open circles are the raw data points from patients (*n* = 14 in each group; 28 patients total). The solid black line is the mean concentration-time profile of liposomal amphotericin B for a patient weighing 68 kg. The broken lines in both panels represents the 5th and 95th percentile for the simulated population.

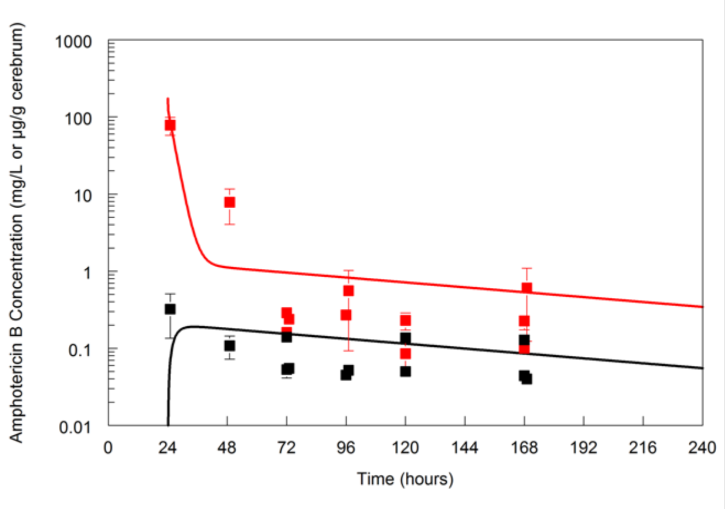

Figure 3 (b). The pharmacokinetics of liposomal amphotericin B in murine plasma (red) and cerebrum (black) in cohorts of mice infected with Cryptococcus neoformans receiving L-AmB 20 mg/kg SINGLE DOSE i.v. The terminal half-life in the plasma and cerebrum is circa 133 hours.

Although L-AmB is recommended as a first-line agent for treating cryptococcal meningitis in several national guidelines[26,27], there is uncertainty regarding optimal dosing strategies. A randomised controlled trial of liposomal L-AmB 3 mg/kg/d vs. 6 mg/kg/d (both for 14 days) vs. amphotericin B deoxycholate showed no differences between any of these regimens[24], and 3 mg/kg/d is widely used as the standard dose. However murine models suggest dosing of 3 mg/kg/day may be sub optimal[18]. Further evidence that this may be the case comes from our recently completed phase II Ambition study[28,29] which was performed with the primary objective of determining the Early Fungicidal Activity (EFA) of three alternative schedules of intermittent high dose L-AmB in comparison with standard daily L-AmB for induction therapy for HIV-associated cryptococcal meningitis to select the optimal short-course L-AmB regimen for this current study. Eighty participants were recruited at sites in Botswana and Tanzania and randomised to one of four treatment arms: (i) L-AmB 10 mg/kg day 1 (single dose); (ii) L-AmB 10 mg/kg day 1, L-AmB 5 mg/kg day 3 (two doses); (iii) L-AmB 10 mg/kg day 1, L-AmB 5 mg/kg days 3, and 7 (three doses). The control arm was standard 14-day L-AmB (3mg/kg/d). All were given with high dose fluconazole 1200mg/day[30,31]. This initial phase II study was stopped by the DMC at the pre-planned interim analysis as the primary endpoint had been reached with the recommendation that the trial proceed onto the current clinical endpoint phase III trial using single dose L-AmB. The three high-dose short-course L-AmB regimens were all extremely well tolerated, with only a single grade IV laboratory toxicity occuring during induction therapy, and a total of seven grade III and no grade IV adverse events asociated with high dose L-AmB, compared to 33% of patients reporting grade III or IV anaemia in a combined cohort of 368 patients treated in Africa with conventional amphotericin B for 2 weeks[25]. There were no safety concerns with short-course treatment, and no patients receiving short course L-AmB required additional "rescue" L-AmB therapy. Overall mortality in the study was 29% at 10 weeks, comparing very favourably with recent trials of amphotericin B deoxycholate based treatments[10], with no significant difference between arms. The primary analysis has shown that the EFA in all 3 short-course high-dose arms is comparable to or greater than the control arm (figure 4).

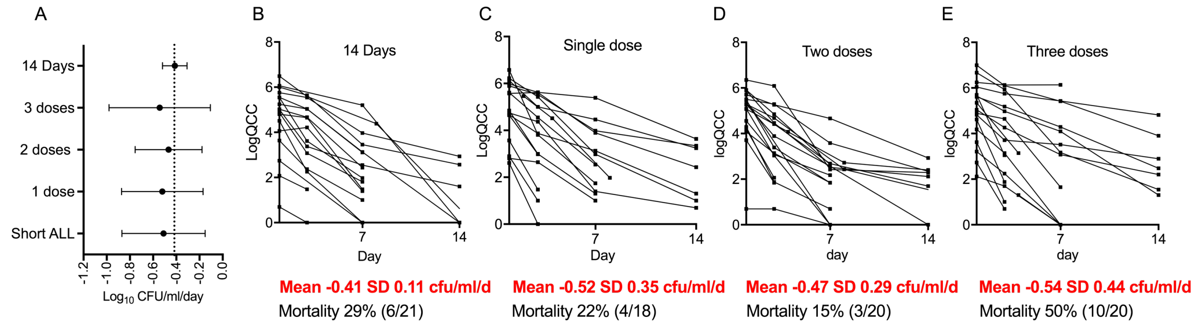

Figure 4. Results from the Ambition-cm Phase-II Trial. *A.* Mean rate of clearance of infection (EFA) with 95% confidence intervals in the four study arms. Short ALL shows the combined data from the three short-course high-dose arms. *B.* Fungal clearance slopes from participants in the control arm. *C. D. E.* Individual fungal clearance slopes from participants in the short-course arms. CFU = colony forming units, QCC = quantitative fungal culture, SD = standard deviation.

All the short course arms high doses of L-AmB were non-inferior in terms of EFA to 14 day doses of low dose L-AmB at the pre-defined non-inferiority (NI) of 0.2 $\log_{10}$cfu/ml/day, with no evidence for any dose response effect with additional L-AmB doses, suggesting maximal fungicidal activity was achieved with a single 10mg/kg dose (Figure 5). This remained the case when the analysis was adjusted for factors that have previously been shown to affect EFA (QCC, Abnormal Mental Status, CD4).

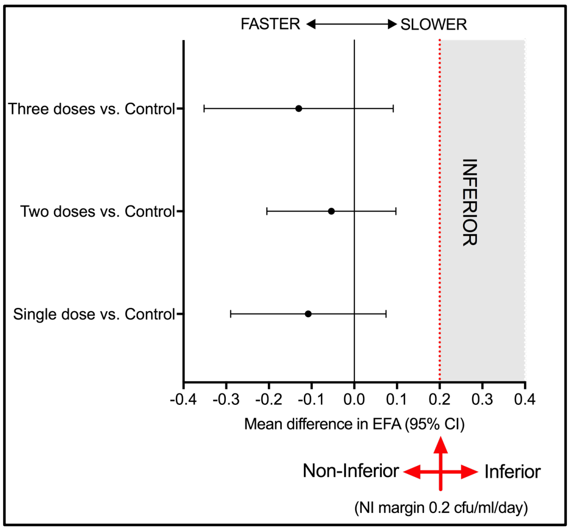

Figure 5. Mean difference in EFA between short course and control regimens with 95% confidence intervals. All short course arms demonstrate non-inferiority to the control arm at the pre-defined non-inferiority (NI) margin of 0.2 cfu/ml/day.

1.5 RISKS AND BENEFITS

Our previous studies[10,30,34], and those of other groups using EFA outcomes[33,35] have confirmed the safety of deriving EFA measurements from lumbar punctures (LPs) performed on days 1, 7 and 14 in patients with HIV-associated CM. In this cumulative experience with over 1500 patients, no serious adverse events have been attributed to additional LPs performed for study purposes. In fact, accumulating evidence suggests that the protocolised measurement of CSF pressure and aggressive management of raised CSF pressures that is possible in EFA studies actually improves outcomes in patients[10,36]. Given the symptomatic relief that often accompanies LP and pressure drainage, patient compliance with repeat LPs is good.

For individuals participating in this study there is a risk that the new treatment regimens could prove inferior to standard therapy. Choosing a regimen based on high quality phase-II data reduces this risk but stringent safety measures including regular clinic review and CSF cultures (see Section 6. Assessments and Follow-up) have been incorporated into the study protocol to ensure that any patients failing to respond to short course therapy are identified and can receive a full course of rescue therapy with standard daily dosed AmB plus flucytosine if required. The Data Monitoring Committee (DMC) will closely monitor the trial.

A further risk to study participants is potential toxicity due to the high dose L-AmB. However high dose L-AmB has been used extensively in haematology and oncology patients with invasive fungal infections, in patients with visceral leishmaniasis, and in 59 of our phase-II study patients with CM. In our phase II study there were no safety concerns with the high dose short course arms (see Section 1.4). Toxicity in the study participants will be closely monitored and regularly reported to the DMC. We anticipate that it is much more likely that study participants will benefit from the reduced toxicity profile of the shorter course L-AmB regimen compared to standard amphotericin deoxycholate, and may also be able to benefit from shorter hospital stays and avoid the complications associated with multiple intravenous infusions.

The study will generate important knowledge, which has the potential for a large impact on guidelines and policy, both by reducing the 10-week mortality of cryptococcal meningitis in the majority of African centres still relying on fluconazole monotherapy from approximately 60%[13,37] to the 25-35% seen with AmB-based combinations[11,12], and by facilitating effective treatment, reducing duration of

hospital admissions, and lowering the high burden of drug related side effects currently seen in centres using conventional D-AmB based therapy.

SELECTION OF SITES/CLINICIANS

The Trial Management Group has overall responsibility for site and investigator selection.

2.1 SITE/INVESTIGATOR INCLUSION CRITERIA

Trial recruitment will take place at 6 African partner sites.

- Mitchells Plain District Hospital and Khayelitsha Hospital, Cape Town, South Africa (University of Cape Town)
- Princess Marina Hospital, Gaborone, Botswana (Botswana-Harvard Partnership)
- Parirenyatwa Central Hospital, Harare, Zimbabwe (University of Zimbabwe)
- Queen Elizabeth Central Hospital, Blantyre, Malawi (College of Medicine/ MLW)
- Kamuzu Central Hospital, Lilongwe, Malawi (UNC Project)
- Infectious Diseases Institute, College of Health Sciences, Makerere University, Uganda (IDI)

The trial sites build on prior successful collaborations, and have been selected due to their proven ability to successfully execute clinical trials of HIV-associated CM, and to reflect a spectrum of African health care settings, ranging from Cape Town and Gaborone where amphotericin B based therapy is the current standard of care, to Uganda where fluconazole monotherapy is often the only available treatment. The phase-II study was conducted in Gaborone, with a trained team in place, enabling rapid commencement of recruitment at this site on commencement of the phase-III trial.

The study will be performed over 4 years, with planned commencement in January 2017. The first year (2017) will involve development of study documentation, site preparation, and applications for ethical and regulatory approvals. Patient recruitment will take place over the latter part of year 1, years 2, 3, and the first part of year 4 (36 months) allowing recruitment of an average of 142 patients per study site at a rate of roughly one participant per week (a conservative estimate based on prior experience at the proposed sites in the Ambition Phase-II, ACTA, and COAT trials[35]). Breakdown by site is as follows: Blantyre 230 overall, 6/month[38]; Cape Town 80 overall, 2-3/month[12,35]; Gaborone 90 overall, 2-3/month[39]; Harare 230 overall, 6/month[40]; Lilongwe 110 overall, 3/month[14,37], Kampala 110 overall, 3/month[35,41,42].

Once a site has been identified as being compliant with the inclusion criteria (and not excluded), the trial team will provide the site with a copy of this protocol, a trial summary, the Summary of Product Characteristics (SPC), and the Investigators' Brochures (IB).

To participate in the AMBITION-*cm* trial, investigators and clinical trial sites must fulfil a set of basic criteria that have been agreed by the AMBITION-*cm* Trial Management Group (TMG) and are defined below.

Sites where a previous serious protocol breach has occurred will be visited and thoroughly reviewed before allowing participants to enter the trial.

Those centres that meet the criteria will be issued with the AMBITION-*cm* master file documentation, the EDCTP Consortium Agreement, and EDCTP Accession Forms.

2.1.1 PI'S QUALIFICATIONS & AGREEMENTS

1. The investigators should be qualified by education, training, and experience to assume responsibility for the proper conduct of the trial at their site and should provide evidence of such qualifications through an up-to-date curriculum vitae and/or other relevant documentation requested by the Sponsor, the research ethics committee (REC), the institutional review board (IRB), and/or the regulatory authority(ies).

2. The investigator should be thoroughly familiar with the appropriate use of the investigational product, as described in the protocol, the product information and in other information sources provided by the Sponsor.

3. The investigator should be aware of, and should comply with, the principles of ICH GCP and the applicable regulatory requirements. A record of GCP training should be accessible for all investigators.

4. The investigator/site should permit monitoring and auditing by the Sponsor, and inspection by the appropriate regulatory authority(ies).

5. The investigator should maintain a delegation log of appropriately-qualified persons to whom the investigator has delegated significant trial-related duties.

6. The investigator should sign an investigator statement, which verifies that the site is willing and able to comply with the requirements of the trial.

2.1.2 ADEQUATE RESOURCES

1. The investigator should be able to demonstrate a potential for recruiting the required number of suitable subjects within the agreed recruitment period (that is, the investigator regularly treats the target population).

2. The investigator should have sufficient time to properly conduct and complete the trial within the agreed trial period.

3. The investigator should have available an adequate number of qualified staff and adequate facilities for the foreseen duration of the trial to conduct the trial properly and safely.

4. The investigator should ensure that all persons assisting with the trial are adequately informed about the protocol, the investigational products, and their trial-related duties and functions.

5. The site should have sufficient data management resources to allow prompt data return to the co-ordinating centre (refer to the Data Management Plan for timelines). Sites that have previously participated in trials coordinated by the same centre should have a proven track record of good data return.

2.1.3 SITE ASSESSMENT

Each selected clinical trial site must complete the AMBITION-*cm* EDCTP Consortium Agreement, Accession Form, Signature and Delegation of Responsibilities Log, and staff contact details. The Accession Form verifies that the site is willing, and able to comply with the requirements of the trial. In addition, and in compliance with the principles of ICH GCP, all site staff participating in the trial must complete the Signature and Delegation of Responsibilities Log and forward this to the co-ordinating centre. The co-ordinating centre must be notified of any changes to trial personnel and/or their

responsibilities. An up-to-date copy of this log must be stored in the Trial Master File (TMF) at the site and at the co-ordinating centre.

2.2 SITE/INVESTIGATOR EXCLUSION CRITERIA

Failure to meet the above requirements.

2.3 APPROVAL AND ACTIVATION

The ethics committees/Institutional Review Boards of the participating institutions will be supplied with the names and addresses of all participating site principal investigators. Trial staff at the co-ordinating centre will perform this task; hence it is vital to receive full contact details for all investigators prior to their entering patients.

1. The site should conduct the trial in compliance with the protocol as agreed by the Sponsor and, if required, by the regulatory authority(ies), and which has been approved by the REC and/or IRB.
2. The PI or delegate should document and explain any deviation from the approved protocol, and communicate this with the trial team at the co-ordinating centre.

A list of activated sites may be obtained from the Trial Manager.

2.4 SITE MANAGEMENT

The Cape Town site is led by the highly experienced and long-standing cryptococcal trialist Graeme Meintjes, based at the University of Cape Town, who has been an investigator on numerous cryptococcal treatment trials, and was a lead investigator on the multi-centre trial of ART timing recently published in the NEJM[35]. The Botswana site, based at the Botswana-Harvard Partnership, has successfully conducted a number of clinical trials of CM treatments[33,39,43], recruited patients for the phase-II EFA study, and has an effective study team already in place. The Zimbabwe team led by Professor Chiratidzo Ndhlovu and Dr Admire Hlupeni, based at the University of Zimbabwe, performed and published one of the seminal studies investigating ART timing in CM[40], and is currently performing studies investigating the utility of cryptococcal antigen screening as a strategy to prevent CM. The two proposed Malawian sites (College of Medicine / Malawi-Liverpool Wellcome Trust Clinical Research Programme and UNC-Project Malawi) both have on-going active CM research programmes[14,37,38,44], and have built considerable local expertise in the conduct of cryptococcal clinical trials. The Investigators at the Ugandan site, based at the Infectious Diseases Institute, have recently completed an NIH- funded trial of ART timing after Cryptococcal meningitis and a CDC-funded trial for cryptococcal antigen screening to improve ART survival, and are currently running an NIH and UK Medical Research Council co-funded trial to determine the utility of Sertraline as adjunctive therapy for cryptococcal meningitis[35,41,42]. All sites have agreed to and are fully committed to study participation.

SELECTION OF PATIENTS

There will be **no exceptions** to eligibility requirements at the time of randomisation. Questions about eligibility criteria should be addressed prior to attempting to randomise the participant.

The eligibility criteria for this trial have been carefully considered. The eligibility criteria are the standards used to ensure that only medically appropriate patients are considered for this study. Patients not meeting the criteria should not join the study. For the safety of the patients, as well as to ensure that the results of this study can be useful for making treatment decisions regarding other patients with similar diseases, it is important that no exceptions be made to these criteria for admission to the study.

Participants will be considered eligible for enrolment in this trial if they fulfil all the inclusion criteria and none of the exclusion criteria as defined below.

3.1 PATIENT INCLUSION CRITERIA

1. Consecutive patients aged $\geq$ 18 yrs with a first episode of cryptococcal meningitis (CSF India ink or CrAg test)
2. HIV positive or willing to undertake an HIV test if unknown HIV status
3. Willing to participate in the study or, if unable to consent, has a next of kin who agrees to the patient participating in the study

3.2 PATIENT EXCLUSION CRITERIA

1. Pregnancy (confirmed by urinary or serum pregnancy test) or breastfeeding
2. Previous serious reaction to amphotericin, flucytosine or fluconazole
3. Already taking antifungal treatment at cryptococcal meningitis treatment doses (amphotericin B ≥0.7mg/kg or fluconazole ≥800mg/day) for >48 hours
4. Contraindicated oncomitant medications including: cisapride and the class of antihistamines including terfenadine.
5. HIV negative

3.3 NUMBER OF PATIENTS

850 patients (425 per arm)

850 patients will be enrolled (425 in the control arm and 425 in the short-course L-AmB). The intake will take place over a 3-year period (see **Section 2**), and follow-up will occur for 16 weeks after the start of treatment. The total duration of the study will be 39 months.

3.4 CO-ENROLMENT GUIDELINES

Co-enrolment in previous or future trials is considered in Section 4.3 Co-enrolment Guidelines.

3.5 SCREENING PROCEDURES & PRE-RANDOMISATION INVESTIGATIONS

Patients over 18 years of age with a first episode of cryptococcal meningitis diagnosed based on CSF India ink or cryptococcal antigen test at the local laboratory will be assessed for eligibility for inclusion in the trial. Their details will be recorded in a screening register. Patients will be enrolled into the study based on the criteria of eligibility outlined in **sections 3.1 and 3.2**. For those found to be ineligible the reason for non-inclusion should be recorded on the screening register.

Written informed consent to enter the trial and be randomised must be obtained from participants or, in the case of those lacking capacity to consent, from family/guardians/person with legal responsibility (if appropriate and in keeping with National guidance and regulations), after explanation of the aims, methods, benefits and potential hazards of the trial, and BEFORE any trial-specific procedures are performed or any blood is taken for the trial (see Consent Forms).

Patients with altered mental status who are unable to consent will be enrolled into the study if their next of kin gives informed consent or assent (in keeping with appropriate National guidance and regulations) on their behalf. As soon as the patient's mental status improves consent will be obtained as above, with care taken to ensure they understand that they are free to withdraw from the study and if they do so this will not jeopardise their future care.

It must be made completely and unambiguously clear that the participant (or guardian) is free to refuse to participate in all or any aspect of the trial, at any time and for any reason, without incurring any penalty or affecting their access to the standard treatment available at the recruiting site (or that of their relative).

Original signed consent forms must be kept by the investigator and documented in the electronic case report form eCRF, a copy given to the participant or family, and a copy placed in the participant's medical notes.

REGULATION & RANDOMISATION

Patients will be randomised individually using a computer-generated programme. Randomisation will be stratified by site.

4.1 RANDOMISATION PRACTICALITIES

Further details on the process of randomisation can be found in Section 9.1 - Method of Randomisation.

RANDOMISATIONS

To randomise:
Upon confirmation of eligibility criteria and completion and entry of the informed consent details, the electronic data capture system (EDC) will select the next available randomization slot and present the details of the selection to the Study Doctor / Nurse on screen.

Tel: +267 390 2671 ext. 2210; 2230; 2203 or email ambition-support@bhp.org.bw for assistance. Note: Randomisation is stratified by site ONLY. The first digit of the randomisation code is the site prefix.

4.2 RANDOMISATION CODES & UNBLINDING

Randomisation codes are as detailed above. Randomisation is stratified by site. The trial is open label.

4.3 CO-ENROLMENT GUIDELINES

Patients may not be enrolled in any other studies investigating treatment of cryptococcal meningitis, or which may impact on the management of their cryptococcal meningitis as laid down in this protocol. Enrolment in other trials must be discussed with and approved by the TMG and will be at the discretion of the local PIs and under the direction of the site specific local Research Ethics Committees.

TREATMENT OF PATIENTS

5.1 BASELINE ASSESSMENT

i) Clinical:

Age, Sex
Significant past medical history, Drug history, time on ART and adherenece assessment (if on ART)
Symptoms and duration
Physical examination

ii) Laboratory:

- Full blood count (FBC), urea, creatinine, electrolytes, alanine transaminase (ALT)
- HIV serology – if status not already known (with documentation of result)
- CD4 count, and VL if on ART as is routine at the site(s)
- Pregnancy test – urine or serum in women of reproductive age
- CSF: opening pressure, cell count and differential, protein, glucose,
  India ink, quantitative fungal culture, routine culture, cryptococcal antigen

5.2 ANTIFUNGAL DRUG TREATMENT

5.2.1 DRUG REGIMENS

Patients will be randomised to one of two study regimens:

1. L-AmB 10 mg/kg day 1 (single dose) given with fluconazole 1200mg/day plus flucytosine 100mg/kg/d for 14-days

2. Amphotericin B deoxycholate 1 mg/kg/d for 7-days given with flucytosine 100mg/kg/d followed by fluconazole 1200mg/day for 7-days (standard dose, "control arm")

All patients receive fluconazole 800 mg/d to 10 weeks (provision will be ensured throughout the trial), and 200 mg/d thereafter (as per national guidelines at the site). ART will be commenced 4 – 6 weeks after initiation of antifungal therapy.

Initial intensive phase (first 2 weeks): Patients will be admitted to hospital for the initial intensive phase of treatment, with all patients managed as inpatients for at least 7-days. Individuals may be discharged after day 7 and prior to day 14, in which case treatment will be given under close outpatient supervision during the second week of treatment, ensuring compliance to the trial intervention and facilitating close clinical and laboratory monitoring.

Continuation phase (second 8 weeks): Patients will be followed in the outpatient clinic and given medication to take at home.

5.2.2 Source of drugs

Fluconazole (Diflucan, Pfizer) will be obtained locally through the Pfizer nationwide donation programme or purchased from a WHO qualified generic manufacturer should donated supplies be insufficient. Flucytosine will be sourced from Meda Pharmaceuticals Inc. Amphotericin B deoxycholate will be sourced from Bristol-Myers Squibb (BMS). L-AmB (Ambisome) is sourced by Gilead Sciences Europe Ltd. Drugs will be shipped directly to the sites following approval from the relevant regulatory authority.

5.2.3 Dispensing

The drugs will be stored in the pharmacy, clearly labelled as trial medication, and segregated from other pharmacy stock. The trial staff will collect the amounts needed from the pharmacy. Detailed records of drugs dispensed and received will be maintained by both the pharmacist and trial staff.

5.2.4 Dose Modifications, Interruptions & Discontinuations

See manufacturer's latest Summary of Product Characteristics (SPC) for L-AmB, Amphotericin, Flucytosine, and Fluconazole for dose adjustments in renal/hepatic insufficiency and management of expected adverse events. The Ambition trial treatment document may also be referenced in the absence of an internet connection.

- http://www.medicines.org.uk/emc/medicine/1236
- http://www.medicines.org.uk/emc/medicine/559
- https://www.drugs.com/pro/flucytosine.html
- http://www.cipla.co.za/wp-content/uploads/2013/10/sep_cipla-fluconazole-50mg.pdf

5.2.4.A Stopping Drug Early

Discontinuation criteria are considered in Section 5.4 - Protocol Treatment Discontinuation

5.2.5 Accountability & Unused Drugs/Devices

Drug stocks will be regularly monitored and the remaining stocks checked against the amount dispensed.

The investigator/pharmacist/investigational drug storage manager must maintain records of the drugs delivery to the trial site, the inventory at the site, the use by each patient.

These records will include dates, quantities, batch/serial numbers, expiry ('use by') dates, and patient's trial numbers. The investigator/pharmacist/investigational drug storage manager will maintain records that document adequately that the patients were provided the doses specified by the protocol and reconcile all investigational product(s) received from the sponsor.

5.2.6 Compliance & Adherence

The first 7-days of the two week induction treatment will be given in a directly observed hospital setting. Patients may be discharged from day 7 at which point they will remain under close outpatient supervision during the second week, ensuring compliance to the trial intervention. Our experience with cryptococcal treatment trials using some of the same sites has shown loss to follow-up at 10 weeks of less than 2% (ACTA study; ISRCTN: 45035509). To obtain accurate and complete follow-up data for 16 weeks after the start of treatment, telephone numbers and addresses will be collected for patients and relatives, regular telephone contact will be made, and financial assistance will be provided to cover travelling expenses. If a patient fails to attend outpatient clinic visits, the study team will visit the home address to asses vital status and if alive make every effort to persuade the patient to attend and continue antifungal and antiretroviral treatment.

Management of raised CSF opening pressure will be clearly documented in the case record forms and any deviation from standard operating procedures will be recorded and justified. Timing of ART inititation or restarting will be documented in CRFs.

Please refer to the trial specific pharmacy SOPs and SPC document for advice on prescribing, IMP formulation, storage, labelling, temperature monitoring, administration, cautions, contra-indications, accountability and returns information.

5.3 OTHER TREATMENT

5.3.1 PREHYDRATION AND ELECTROLYTE SUPPLEMENTATION

To minimise the risk of Amphotericin based therapy induced toxicities all patients will routinely receive 1 litre of Normal Saline with 20mmol of KCl infused over 1-2 hours before the L-AmB/D-AmB infusion. This reduces the risk of renal impairment and hypokalaemia. All patients will also be prescribed oral potassium and magnesium supplementation, with 2 tablets twice daily of potassium chloride (Slow-K, 600mg, 8mmol K/tab) and 2 tablets once daily of magnesium chloride (Slow-Mag 535mg, 5.33mmol Mg/tab) for the duration of Amphotericin based therapy and for two days post discontinuation of L-AmB dosing with additional IV or oral supplementation if potassium or magnesium levels drop below normal ranges.

5.3.2 MANAGEMENT OF RAISED CSF OPENING PRESSURE

Patients with an opening pressure greater than 30 cm will have daily lumbar punctures to remove CSF consistent with guidelines (see Standard Operating Procedure).

5.3.3 ANTIRETROVIRAL TREATMENT

Patients who are ART naïve will start ART 4-6 weeks after initiation of antifungal therapy. The ART drugs used will conform to the local treatment protocols in use. Timing of ART is in accordance with local and international guidelines[26,45,46], and is the earliest time-point currently accepted as good clinical practice. Patients who have been exposed to ART will be managed as per a standardised operating procedure including viral load testing, adherence assessment, enhanced adherence counselling, and resistance testing in line with National and site specific guidelines. Treatment switches to second line therapy in individuals failing first line therapy due to suspected drug resistance will be made 4-6 weeks after initiation of antifungal therapy. ART experienced individuals who are not virologically suppressed at baseline will have a repeat viral load at three months after any treatment switch or adherence intervention to ensure either re-suppression or appropriate treatment failure management.

5.4 PROTOCOL TREATMENT DISCONTINUATION

In consenting to the trial, patients are consenting to trial treatment, trial follow-up and data collection. However, an individual patient may stop treatment early or be stopped early for any of the following reasons:

- Fulfilment of an early withdrawal criterion. (The early withdrawal criterion for this trial are an ALT>5 times upper limit of normal i.e. > 200 IU/mL, absolute neutrophil count of <500 x 10^6/L; or platelets <50,000x10^6/L on baseline blood testing, and Cryptococcal meningitis not confirmed – see Trial Treatment Document).
- Unacceptable toxicity or adverse event (e.g. development of severe hepatic or renal dysfunction on treatment. For full details see the Trial Treatment Document)

- Intercurrent illness that prevents further treatment
- Any change in the patient's condition that justifies the discontinuation of treatment in the clinician's opinion
- Inadequate compliance with the protocol treatment in the judgement of the treating physician
- Withdrawal of consent for treatment by the patient

As the patient's participation in the trial is entirely voluntary, they may choose to discontinue the trial treatment at any time without penalty or loss of benefits to which they are otherwise entitled. Although the patient is not required to give a reason for discontinuing their trial treatment, a reasonable effort should be made to establish this reason while fully respecting the patient's rights.

Patients should remain in the trial for the purpose of follow-up and data analysis (unless the patient withdraws their consent from all stages of the trial). If a patient is withdrawn from follow-up, refer to Section 6.6 - Early Stopping of Follow-up.

Data will be kept and included for patients who stop follow-up early as long as consent for this is not withdrawn.

5.5 TREATMENT DATA COLLECTION

Details of the dose, timing and route of administration of all study medication will be recorded in the eCRFs and treatment charts of study patients by the study team, who will administer or directly observe the administration of all study medication during hospitalisation.

5.6 NON-TRIAL TREATMENT

All non-trial treatment taken by the patient will be recorded at enrolment, at follow-up, and in the event of an SAE occurring.

5.6.1 Medications Permitted

Drugs not known to be contraindicated with the trial drugs will be permitted.

5.6.2 Medications Not Permitted

Fluconazole is contraindicated in combination with cisapride and the class of antihistamines including terfenadine. Other drugs (including cisapride, astemizole, pimozide, quinidine and erythromycin) that have potential to prolong the QT interval should be avoided if possible during the first 2 weeks of treatment.

5.6.3 Medications to be Used With Caution

Rifampicin – May reduce fluconazole levels.

5.6.4 Treatment After Trial Event

Treatment will be at the discretion of the responsible physician.

5.7 CO-ENROLMENT GUIDELINES

Co-enrolment in previous or future trials is considered in Section 4.3 - Co-enrolment Guidelines.

ASSESSMENTS & FOLLOW-UP

During the two-week induction phase patients will initially be admitted and clinical responses monitored daily. Patients may be discharged from day 7, in which case they will receive close outpatient follow-up with daily calls, blood tests as described in the protocol, until they receive a clinic review at day 14. Ongoing outpatient follow-up will be at 4, 6, 8 and 10 weeks. At 10 weeks patients will be transferred into the local ART clinic for continuing management. A single telephonic contact at 16 weeks to assess vital status and simple disability score will be made.

6.1 TRIAL ASSESSMENT SCHEDULE

i) Clinical response:
Clinical response will be monitored daily for the first 2 weeks, then in follow-up 4, 6, 8 and 10 weeks after start of therapy. A single telephonic follow-up to ascertain vital status and simple disability score will be made at week 16. Every effort will be made (for example with mobile telephone calls and financial help with travelling expenses) to obtain accurate and complete follow-up data for 10 weeks after the start of treatment. Particular attention will be paid to the possibility, in ART-naïve patients, of immune reconstitution reactions after starting ART.

ii) Laboratory data:
Day 3, 5, 7, 10, 12, 14: Creatinine, Electrolytes
Day 7, 14: Full blood count, ALT
Week 4 (clinic): Full blood count, Creatinine, Electrolytes, ALT

At the time of monitoring blood tests, and at additional times (see schedule below), an extra 2.5 ml of blood will be taken for PK studies.

Days 7, 14: Follow up CSF examinations (performed by study team) for: opening pressure, quantitative fungal culture, CSF for drug levels, immune parameters.

TABLE 6.1. Event Schedule	Screening	Week 1							Week 2							Wk 4	Wk 6	Wk 8	Wk 10	Wk 16
**Study Day**	≤D0	D1	D2	D3	D4	D5	D6	D7	D8	D9	D10	D11	D12	D13	D14					
**Consent forms**																				
PIS and signed consent	X	X																		
**Follow up**																				
Screening and randomisation	X	X																		
Clinical review		X	X	X	X	X	X	X	X	X	X	X	X	X	X					
Outpatient follow-up																X	X	X	X	
Week 16 telephone																				X
**Clinical labs**																				
HIV testing		X																		
Pregnancy Test (Urine/Serum) (2)		X																		
Full Blood Count		X						X							X	X				
CD4 count		X																		
ALT		X						X							X	X				
Urea, creatinine and electrolytes		X		X		X		X			X		X		X	X				
**Clinical evaluation**																				
Chest X-ray (1)		X																		
**CSF**																				
Opening pressure		X						X							X					
Cell count and differential*		X																		
Protein, glucose*		X																		
Routine culture *		X																		
India ink examination* (3)		X																		
Cryptococcal antigen* (3)		X																		
Quantitative fungal culture		X						X							X					
CSF Drug levels		X						X							X					
Immune parameters		X						X							X					

*Part of routine care. 1. If clinically indicated. 2. For women of childbearing age. 3. India ink or cryptococcal antigen required for inclusion

6.2 PROCEDURES FOR ASSESSING EFFICACY

All deaths during the study period will be recorded. Cause of death will be clinically ascertained by the study physicians (patients will not receive post-mortems). Mortality by treatment group will be analysed with all-cause mortality within the first 10 weeks as the primary endpoint. Cryptococcal clearance rates will be calculated using summary statistics for each patient: the rate of decrease in $\log_{10}$ CFU per ml CSF per day derived from the slope of the linear regression of $\log_{10}$ CFU against time for each patient. A linear regression model will be used to compare mean rates of decline or early fungicidal activity (EFA) for each experimental treatment, giving summary differences with 95%CI and significance levels[32,47]. We will adjust analyses for potential confounding factors, including baseline fungal load. Disability at 10 weeks will be assessed using two simple questions and a modified Rankin score (Table 1).

Table 6.2.

**The two simple questions**		
Does the patient require help from anybody for everyday activities? *(For example eating, drinking, washing, brushing teeth, going to the toilet.)*	Yes/no	Yes = Poor outcome
Has the illness left you with any other problems?	Yes/no	Yes = Intermediate outcome
		No = Good outcome
**The modified rankin scale**		
*Grade**	*Description*	
0	No symptoms	
1	Minor symptoms not interfering with lifestyle	
2	Symptoms that lead to some restriction in lifestyle, but do not interfere with the patients' ability to look after themselves	
3	Symptoms that restrict lifestyle and prevent totally independent living	
4	Symptoms that clearly prevent independent living, although the patient does not need constant care and attention	
5	Totally dependent, requiring constant help day and night	

*Grade 0: Good outcome, Grade 1 or 2: Intermediate outcome, Grade 3-5: poor outcome.

6.3 PROCEDURES FOR ASSESSING SAFETY

There is considerable experience with L-AmB doses up to 10 mg/kg/d. In a trial of treatment for filamentous fungal infections[23] with 100 patients per arm, daily dosing at 10 mg/kg/d compared with 3 mg/kg/d, for 14 days, was associated with increased nephrotoxicity. However, rates of doubling of creatinine and of hypokalaemia were still only those seen with the use of conventional D-AmB formulation 0.7 mg/kg/d in cryptococcal meningitis[11]. Single dose and intermittent L-AmB at up to 10 mg/kg/d has been very well tolerated[19,20,22,29]. Serum creatinine and potassium will be monitored at predetermined intervals to ensure any adverse effects are recognised early and appropriate management undertaken. .

Proportions of patients in different arms suffering clinical and laboratory-defined side effects will be compared as well as the mean percent change from baseline of laboratory values in the treatment groups. Mortality by treatment group will be analysed by summary statistics and time to event analyses, unstratified and stratified for possible confounding factors. The frequency and severity of any immune reconstitution reactions, the factors associated with occurrence of reactions and the frequency of further AIDS-related illnesses will also be determined. These parameters will be reviewed during the study by the Independent Data Monitoring Committee (IDMC – see Section 9.4)

Throughout the study patients will be closely monitored for signs and symptoms of drug toxicity. All toxicities leading to temporary or permanent discontinuation of the study therapy, and all Grade 3 or 4 toxicity effects will require thorough investigation with relevant clinical and laboratory tests as clinically indicated. These should be repeated until final resolution or stabilization of the toxicity. All symptoms and laboratory findings will be graded according to severity using the modified Division of AIDS toxicity criteria[48].

In the event that the day 7 lumbar puncture identifies an increase in colony forming units from baseline this must be reported as an SAE. The co-ordinating centre will be responsible for managing this situation on a case-by-case basis.

6.4 PROCEDURES FOR ASSESSING PHARMACOKINETICS

Plasma samples to describe the PK of liposomal amphotericin B, and enable the PK-PD of liposomal amphotericin B for CM and the impact of PK variability on outcome to be described, will be collected at the end of infusion, then 3, 6, 8 and 24 hours in a substudy of patients at two Ambition Trial recruitment sites. This will likely be Botswana where PK sampling as described has already been undertaken in the phase II study, plus Blantyre.

In addition, in all Ambition trial patients, two samples will be collected at 6 and 24 hours post infusion for L-AmB levels. The serum pharmacokinetics of fluconazole will also be described. A portion (0.5 mL) of the CSF sample obtained for quantitative counts will be reserved to measure fluconazole concentrations, and thereby estimate the extent of penetration of fluconazole into the CSF. We will not attempt to measure concentrations of amphotericin B in CSF as the drug is concentrated in the meninges and brain and cannot be reliably detected in CSF.

The PK-PD data will be modelled using a population methodology, using the program Pmetrics. The same structural mathematical model as previously described by us and applied to rabbit data will be used. The PK-PD model will enable an in depth understanding of the persistence of amphotericin B within the central nervous system and the resultant antifungal effect. Monte Carlo simulation will enable further insights into the regimen(s) that are associated with maximal antifungal activity including dose of liposomal amphotericin B and the maximum interval that can be utilized in clinical settings.

The ability to fit large PK-PD mathematical models to data from patients will require advanced pharmacometric skills that include PK modelling, modelling of tissue penetration, population PK, pharmacodynamics and Monte Carlo simulation. The co-administration of liposomal amphotericin B with fluconazole will also provide the ability to examine for and model any possible interaction between the two agents. This will require separate fluconazole PK-PD datasets that are currently being analysed by the Antimicrobial Pharmacodynamics and Therapeutics Laboratory in Liverpool. APT has a long track record in fitting combination PK-PD models to define the effect (i.e. synergy, antagonism, additivity) of combination chemotherapy.

6.5 OTHER ASSESSMENTS

6.5.1 ECONOMIC ANALYSES

The objectives of the economic analysis are to estimate the cost consequences and the cost-effectiveness of short-course L-AmB treatment, compared to current care in five country settings across Sub-Sahara Africa, and to inform policy makers on the attractiveness of the new regimen. The expectation is that the short course:

1. Will show a zero net societal cost change or that there will be societal cost savings from the short L-AmB treatment, reducing hospitalization costs over the trial time horizon.
2. Will be cost-effective in terms of societal and service cost of life years saved within the diversity of Sub-Saharan settings when compared to historical cohorts receiving the current standard treatment regimen in each country.
3. That the economic evidence will support wide-scale implementations.

The health service costs of the regimens will be compared, and used along with the primary endpoint data to perform cost-effectiveness modeling at the Liverpool School of Tropical Medicine.

Information on resource and unit costs will be collected at patient-level through the CRFs, as part of the trial, and through two additional separate country costing studies. Firstly, an empirical cost-consequence analysis will take place, using empirical individual patient data on societal resource use and unit cost based on the results from the costing studies in a total of five countries. Both the societal and health care perspective are chosen, and health service costs including, household cost, treatment cost and hospitalisations in both arms will be compared over the trial period. Next, the costing information will be used in a TreeAge cost-effectiveness model that computes health service cost and number of life years survival. Here the two trial arms will be compared to the results from a recent historical cohort. This approach avoids the stochastic fallacy and will determine if short-course high-dose L-AmB will be as or even more cost-effective compared to the current standard of care.

6.5.2 EVALUATION OF THE UTILITY OF BASELINE CRYPTOCOCCAL ANTIGEN TITRE ASSESSMENT BY SEMI-QUANTITATIVE CRAG TESTING AND AN EVALUATION OF THE UTILITY OF DIAGNOSTIC QUANTITATIVE PCR

There are no individualized treatment protocols for the management of cryptococcal meningitis. However, organism load at baseline, as assessed by CFU counts or antigen titre, is known to be a powerful prognostic factor. It is conceivable that an abbreviated course of L-AmB may give equivalent or superior (as a consequence of improved safety profile) results to daily conventional AmB at low, but not at the highest baseline antigen titres. A newly developed point of care, lateral flow, semi-quantitative cryptococcal antigen test is now available from Institut Pasteur and Biosynex. We will use this semi-quantitative test in real time to determine antigen titre at baseline, in blood and CSF, and compare results to the currently established point of care test from IMMY. Secondary trial analyses will include the association of baseline titre with outcome (expected to be strongly correlated); but also exploration of the possibility of a differential treatment response between arms according to baseline titre. If such a differential response was observed this sub-study could provide the rationale for and demonstrate the means for individualized treatment, based on a rapid assessment of antigen load. A novel diagnostic quantitative PCR (DNA and RNA) tool will be also be used in each treatment arm and correlated with quantitative culture counts. We aim to estimate the fungal load and fungal viability in blood and CSF at baseline using the PCR in addition to fungal load kinetics. The objective will be to develop a practical alternative to time consuming quantitative cultures in order to improve detection of fungaemia and fungal burden and to identify the best fungicidal treatments in this and subsequent research studies.

6.5.3 VIRULENCE AND IMMUNOLOGICAL STUDIES

In prior studies, we have used patient peripheral blood mononuclear cells (PBMC) and CSF samples and linked clinical baseline and outcome data to better understand the association of systemic and site-of-infection host immune-parameters with outcomes, including survival and the development of

IRIS[10,49-51]. Immune-active adjunctive therapies remain an important goal, given the outcomes achievable even with the best current antifungal drugs and the paucity of new drugs under development. Given the size of this phase-III trial, similar sub-studies will be developed, which will have much greater power, and add value to the main study. Exploration of differences in immune responses between patients presenting after initiating ART compared to those who are ART-naïve will help define the immunopathology of “unmasking” immune reconstitution inflammatory syndrome, and potentially guide future host-directed therapies to improve outcomes among this increasingly important patient group. Blood will also be collected for sub-studies determining how polymorphisms in immune response genes relate to susceptibility to and outcomes from cryptococcal meningitis. This work builds on earlier work from a South African cohort demonstrating polymorphisms in genes associated with intracellular pathogen processing, particularly macrophages, are associated with the development of cryptococcal infection. Samples from this large cohort will provide the power to definitively examine these associations. CSF profiling is feasible across sites, whereas PBMC collection will be restricted to smaller numbers of patients at sites with facilities for and experience in PBMC isolation (Blantyre, Harare, Gaborone, Cape Town). Of note, facilities to perform the detailed flow-cytometric analyses required for future analyses are available at several of the study sites, including Blantyre, Harare and Cape Town. Wherever materials are collected for genetic analysis this will be clearly explained on the consent form, and patients will indicate specific consent to this analysis.

Cryptococcal isolates will be collected for studies on *C. neoformans* evolution and virulence. Our prior studies have begun to define pathogen variation that impacts clinical outcome and started to give more detailed insight into *C. neoformans* evolution[52]. These studies however have been limited in power. The large number of patients in AMBITION-cm will provide for definitive assessment of these associations. Collection and storage of all isolates is feasible and will be incorporated into this work package for future isolate whole genome sequencing, given that sequencing technologies continue to improve and costs reduce.

Note: the sub studies will not interfere with either enrolment or the key outcomes from the main phase III trial. *Analyses will be performed at the recruiting sites or involve technology and skills transfer to the recruiting sites, and will have leadership from both African and European Partners.*

6.6 EARLY STOPPING OF FOLLOW-UP

If a patient chooses to discontinue their trial treatment, they should always be followed up providing they are willing, that is, they should be encouraged to not leave the whole trial and agree to week 10 outcome assessment and week 16 telephonic follow-up. If they do not wish to remain on trial follow-up, however, their decision must be respected and the patient will be withdrawn from the trial completely. The trial team should be informed of this in writing using the appropriate documentation. Patients stopping early have a negative impact on a trial's data. We will ensure patients have all relevant information about the trial so that they can make an informed decision to stop or continue, as they wish.

If the medical data collected during the patient’s participation in the trial are kept for research and analysis purposes, they will be fully anonymised as with all patient data. Consent for future use of stored samples already collected can be refused when leaving the trial early (but this should be discouraged and should follow a discussion).

Patients may change their minds about stopping trial follow-up at any time and re-consent to participation in the trial.

Patients who stop trial follow-up early will not be replaced.

6.7 PATIENT TRANSFERS

If a patient moves from the area, every effort should be made for the patient to be followed up telephonically and for appropriate medical follow-up and treatment to be arranged locally. In Malawi patients may be transferred between sites in Lilongwe and Blantyre.

6.8 LOSS TO FOLLOW-UP

Every effort will be made (for example with mobile telephone calls and financial help with travelling expenses) to obtain accurate and complete follow-up data for at least 10 weeks after the start of treatment. Follow-up will be performed according to this protocol, and outpatient follow-up will be identical in both trial arms. If a patient fails to attend out-patient clinic visits, the research nurse will visit the home address and make every effort to persuade the patient to attend and continue antifungal and antiretroviral treatment.

6.9 ASSESSMENTS AT TRIAL CLOSURE

The trial will be considered closed when the last patient has completed 10 weeks' active follow-up in the study and the 16-week telephonic follow-up call, and all follow-up and laboratory reports including repeat viral load testing in ART failure cases have been received.

SAFETY REPORTING

The principles of ICH GCP require that both investigators and sponsors follow specific procedures when notifying and reporting adverse events or reactions in clinical trials. These procedures are described in this section of the protocol. Section 7.1 - Definitions lists definitions, Section 7.3 - Investigator Responsibilities gives details of the investigator responsibilities and Section 7.4 - Sponsor Responsibilities provides information on sponsor responsibilities.

7.1 DEFINITIONS

The definitions of the EU Directive 2001/20/EC Article 2 based on the principles of ICH GCP apply to this trial protocol. These definitions are given in Table 7.1: Definitions.

Table 7.1: Definitions

TABLE	DEFINITION
Adverse Event (AE)	Any untoward medical occurrence in a patient or clinical trial subject to whom a medicinal product has been administered including occurrences that are not necessarily caused by or related to that product.
Adverse Reaction (AR)	Any untoward and unintended response to an investigational medicinal product related to any dose administered.
Unexpected Adverse Reaction (UAR)	An adverse reaction, the nature or severity of which is not consistent with the information about the medicinal product in question set out in the Summary of Product Characteristics (SPC) or Investigator Brochure (IB) for that product.
Serious Adverse Event (SAE) or Serious Adverse Reaction (SAR) or Suspected Unexpected Serious Adverse Reaction (SUSAR)	Respectively any adverse event, adverse reaction or unexpected adverse reaction that:<br>▪ Results in death<br>▪ Is life-threatening*<br>▪ Requires hospitalisation or prolongation of existing hospitalisation**<br>▪ Results in persistent or significant disability or incapacity<br>▪ Consists of a congenital anomaly or birth defect<br>▪ Is another important medical condition***

*The term life-threatening in the definition of a serious event refers to an event in which the patient is at risk of death at the time of the event; it does not refer to an event that hypothetically might cause death if it were more severe, for example, a silent myocardial infarction.

**Hospitalisation is defined as an inpatient admission, regardless of length of stay, even if the hospitalisation is a precautionary measure for continued observation. Hospitalisations for a pre-existing condition, that has not worsened or for an elective procedure do not constitute an SAE.

*** Medical judgement should be exercised in deciding whether an AE or AR is serious in other situations. The following should also be considered serious: important AEs or ARs that are not immediately life-threatening or do not result in death or hospitalisation but may jeopardise the subject or may require intervention to prevent one of the other outcomes listed in the definition above; for example, a secondary malignancy, an allergic bronchospasm requiring intensive emergency treatment, seizures or blood dyscrasias that do not result in hospitalisation or development of drug dependency. Presence of malaria is not an SAE unless the current infection is life threatening.

7.1.1 MEDICINAL PRODUCTS

An investigational medicinal product (IMP) is defined as the tested investigational medicinal product and the comparators used in the study. (EU guidance ENTR/CT 3, April 2006 revision).

Adverse reactions include any untoward or unintended response to drugs. Reactions to an IMP (i.e. L-AmB) or comparator (i.e. amphotericin B) should be reported appropriately.

7.1.2 ADVERSE EVENTS

Adverse Events include:

- An exacerbation of a pre-existing illness
- An increase in frequency or intensity of a pre-existing episodic event or condition
- A condition (even though it may have been present prior to the start of the trial) detected after trial drug administration
- Continuous persistent disease or a symptom present at baseline that worsens following administration of the study treatment

7.1.3 EXEMPTED ADVERSE EVENTS

Adverse Events do not include:

- Medical or surgical procedures; the condition that leads to the procedure is the adverse event
- Pre-existing disease or a condition present before treatment that does not worsen
- Hospitalisations where no untoward or unintended response has occurred, e.g., elective cosmetic surgery, social admissions
- Overdose of medication without signs or symptoms

7.1.4 DISEASE-RELATED EVENTS

Cryptococcal meningitis carries a high mortality, with an expected acute mortality of approximately 30%. All deaths will be reported as adverse events. Expected disease related morbidities such as development of visual impairment or seizures will also be reported as adverse events. The only exception to this is elevated CSF pressure, which will be routinely recorded in CRFs, and not reported as an adverse event.

7.1.5 OTHER STUDY-SPECIFIC REQUIREMENTS

In the event that the day 7 lumbar puncture identifies an increase in colony forming units from baseline this must be reported as an SAE. The co-ordinating centre will be responsible for managing this situation on a case-by-case basis.

7.2 OTHER NOTABLE EVENTS

7.2.1 PREGNANCY

Pregnant women will be excluded from the study. All participants will be offered appropriate contraception advice at enrolment. If a participant is found to be pregnant during the study they will

discontinue the study intervention and be treated by their attending physicians according to local guidelines. Such patients will be followed to the end of their pregnancy and pregnancy outcomes recorded.

7.3 INVESTIGATOR RESPONSIBILITIES

All non-serious AEs and ARs, whether expected or not, should be recorded in the patient's medical notes and reported in the appropriate section of the eCRF Follow-up Form and sent to the co-ordinating centre within the agreed timescale (as part of the monthly reports from each site). SAEs and SARs should be notified to the co-ordinating centre within 48 hours of the investigator becoming aware of the event.

7.3.1 INVESTIGATOR ASSESSMENT

The chief investigators (or a delegate) will evaluate all SAEs received for seriousness, expectedness and causality. Investigator reports of SUSARs will be reviewed and those that are SUSARs identified and reported to the regulatory authorities. The causality assessment given by the local investigator cannot be overruled in the case of disagreement, but both opinions will be provided in subsequent reports.

7.3.1.A Seriousness

When an AE or AR occurs, the investigator responsible for the care of the patient must first assess whether the event is serious using the definition given in Table 7.1: Definitions. If the event is serious **and not only related to disease progression**, then an SAE Form must be completed and the co-ordinating centre notified within 48 hours.

7.3.1.B Severity or Grading of Adverse Events

The severity of all AEs and/or ARs (serious and non-serious) in this trial should be graded using the toxicity gradings in Section 18.3 – Appendix 2 (DIVISION OF AIDS TABLE FOR GRADING THE SEVERITY OF ADULT AND PEDIATRIC ADVERSE EVENTS).

7.3.1.C Causality

The investigator must assess the causality of all serious events or reactions in relation to the trial therapy using the definitions in Table 7.2: Assigning Type of SAE Through Causality. There are five categories: unrelated, unlikely, possible, probable, and definitely related. If the causality assessment is unrelated or unlikely to be related, the event is classified as an SAE. If the causality is assessed as possible, probable or definitely related, then the event is classified as an SAR.

Table 7.2: Assigning Type of SAE Through Causality

RELATIONSHIP	DESCRIPTION	SAE TYPE
Unrelated	There is no evidence of any causal relationship	Unrelated SAE
Unlikely	There is little evidence to suggest that there is a causal relationship (for example, the event did not occur within a reasonable time after administration of the trial medication). There is another reasonable explanation for the event (for example, the patient's clinical condition, other concomitant treatment).	Unrelated SAE

Possible	There is some evidence to suggest a causal relationship (for example, because the event occurs within a reasonable time after administration of the trial medication). However, the influence of other factors may have contributed to the event (for example, the patient's clinical condition, other concomitant treatments).	SAR
Probable	There is evidence to suggest a causal relationship and the influence of other factors is unlikely.	SAR
Definitely	There is clear evidence to suggest a causal relationship and other possible contributing factors can be ruled out.	SAR

If an SAE is considered to be related to trial treatment and drug is stopped or the dose modified, refer to Section 5.2.4 - Dose Modifications, Interruptions & Discontinuations.

7.3.1.D Expectedness

If there is at least a possible involvement of the trial treatment (or comparator), the investigator must assess the expectedness of the event. An unexpected adverse reaction is one not previously reported in the current Summary of Product Characteristics (SPC) or one that is more frequent or more severe than previously reported. The definition of an unexpected adverse reaction (UAR) is given in Table 7.1: Definitions. Please refer to latest SPC for L-AmB for a list of expected toxicities associated with the drugs being used in this trial. If a SAR is assessed as being unexpected, it becomes a SUSAR.

7.3.1.E Notification

The co-ordinating centre should be notified of all SAEs within 24 hours of the investigator becoming aware of the event.

Investigators should notify the co-ordinating centre of all SAEs occurring from the time of randomisation until 30 days after the last protocol treatment administration. SARs and SUSARs must be notified to the co-ordinating centre until trial closure.

7.3.2 Notification Procedure

1. The SAE eCRF must be completed by the investigator (the consultant named on the Signature List and Delegation of Responsibilities Log who is responsible for the patient's care), with due care being paid to the grading, causality and expectedness of the event as outlined above. In the absence of the responsible investigator, the form should be completed by a member of the site trial team. The responsible investigator should subsequently check the SAE eCRF and make changes as appropriate. The initial report must be followed by detailed, follow-up and final reports as appropriate.

The minimum criteria required for reporting an SAE are the trial number and date of birth, name of investigator reporting and why the event is considered serious.

2. The SAE eCRF must be completed on the trial database. Once submitted, the co-ordinating centre is alerted of the submission by an automated email to its dedicated pharmacovigilance email address: ambitionReporting@lshtm.ac.uk.

3. Follow-up: patients must be followed up until clinical recovery is complete and laboratory results have returned to normal or baseline, or until the event has stabilised. Follow-up should continue after completion of protocol treatment if necessary. A further SAE eCRF,

indicated as 'Follow-up' should be completed as information becomes available. Extra, annotated information and/or copies of test results may be provided separately. The patient must be identified by trial number, date of birth and initials only. The patient's name should not be used on any correspondence and should be deleted from any test results.

4. Staff should follow their institution's procedure for local notification requirements.

7.4 SPONSOR RESPONSIBILITIES

The co-ordinating centre is undertaking a number of duties as delegated by the trial sponsor and is responsible for the reporting of SAEs, SUSARs, and other SARs to the regulatory authorities (competent authorities of countries in which the trial is taking place) and the research ethics committees, as appropriate. Medically-qualified staff at the co-ordinating centre and/or the Chief Investigator (or a medically-qualified delegate) will review all SAE reports received. The causality assessment given by the local investigator at the hospital cannot be overruled; in the case of disagreement, both opinions will be provided in any subsequent reports.

Fatal and life-threatening SAEs and SUSARs must be reported to the competent authorities within 7 days of the co-ordinating centre becoming aware of the event; other SUSARs must be reported within 15 days.

The co-ordinating centre will also keep all investigators informed of any safety issues that arise during the course of the trial.

The co-ordinating centre, as delegated by the Sponsor, will submit Annual Safety Reports in the required format to Competent Authorities (Regulatory Authority and Ethics Committee).

Drug companies involved will be notified of reportable events as appropriate. The co-ordinating centre will also provide companies with a copy of the Annual Safety Report in the required format.

QUALITY ASSURANCE & CONTROL

8.1 RISK ASSESSMENT

The Quality Assurance (QA) and Quality Control (QC) considerations have been based on a formal Risk Assessment, which acknowledges the risks associated with the conduct of the trial and how to address them with QA and QC processes. QA includes all the planned and systematic actions established to ensure the trial is performed and data generated, documented and/or recorded and reported in compliance with the principles of ICH GCP and applicable regulatory requirements. QC includes the operational techniques and activities done within the QA system to verify that the requirements for quality of the trial-related activities are fulfilled. This Risk Assessment has been reviewed by the Sponsor and has led to the development of a Monitoring Plan, standard operating procedures and a tailored training schedule for all parties involved in the trial.

8.2 CENTRAL MONITORING AT THE CO-ORDINATING CENTRE

Co-ordinating centre staff will review electronic Case Report Form (eCRF) data for errors and missing data points.

Other essential trial issues, events and outputs will be detailed in the Monitoring and Quality Management Plan that is based on the trial-specific Risk Assessment.

8.3 ON-SITE MONITORING

The frequency, type and intensity for routine monitoring and the requirements for triggered monitoring will be detailed in the Monitoring Plan. This plan will also detail the procedures for review and sign-off. The study may be subject to audit by the London School of Hygiene & Tropical Medicine under their remit as sponsor, the Study Coordination Centre and other regulatory bodies to ensure adherence to GCP.

8.3.1 DIRECT ACCESS TO PATIENT RECORDS

Participating investigators should agree to allow trial-related monitoring, including audits, ethics committee review and regulatory inspections by providing direct access to source data and documents as required. Patients' consent for this must be obtained.

8.3.2 CONFIDENTIALITY

We plan to follow the principles of the UK Data Protection Act (DPA) regardless of the countries where the trial is being conducted. Consent forms will be stored under the supervision of each local PI in a secured office and accessible to trial staff only. The database does not hold personal details as participants are identified by their study number throughout the trial.

STATISTICAL CONSIDERATIONS

9.1 METHOD OF RANDOMISATION

Patients will be randomised individually and randomisation codes will be generated using SAS PROC PLAN via permuted-block randomisation method stratified by site. Block sizes will vary at 4 and 6. Randomisation lists will be created for each site by an independent statistician and each list will be housed on the electronic database system (EDC) for that particular site, and will be inaccessible to trial staff except to randomise the next eligible participant. Randomised allocation for each trial participant will be provided to trial staff by extracting the next available randomisation allocation, obtained from the randomisation list for that site housed on the database. Internally the EDC selects against an electronic randomization list prepared in advance by the Statistician. The EDC guarantees to make the selection in the natural order of the list filtering by study site only. Once a selection is made, the randomization record is tagged with the patient study allocated identifier, date and time of randomization and other EDC system audit values (username, machine name, etc). A tagged record cannot be selected more than once.

It is impractical to blind the study because of the very high doses of short course L-AmB (currently up to 20 drug vials per dose depending on patient weight) used in the intervention arm, compared to the standard amphotericin B deoxycholate (often only a single vial per patient), given daily, in the control arm. Bias will be minimised by the use of an objective clinical endpoint "all-cause 10-week mortality" as the primary outcome. Laboratory technicians performing EFA will be blinded to study arm. The trial statistician will be blinded regarding the treatment code when developing the statistical analysis plan and writing the statistical analysis programmes, which will be validated and completed using dummy randomisation codes.

9.2 OUTCOME MEASURES

Primary:

1. All-cause mortality within the first 10 weeks (non-inferiority)

Secondary:

1. Early Fungicidal Activity derived from Serial LPs on days 1, 7 and 14.
2. Clinical and laboratory-defined grade III/IV adverse events; median % change from baseline in laboratory defined parameters.
3. PK parameters and PK/PD associations of single high dose L-AmB.
4. Health service costs.
5. All-cause mortality within the first 2 and 4 weeks
6. All-cause mortality within the first 10 weeks (superiority)
7. Rates of cryptococcal relapse / IRIS within the first 10 weeks
8. Disability at 10 weeks measured using a simple two question assessment and modified Rankin Score

9.3 SAMPLE SIZE

The current recommended treatment for HIV-associated CM are 7-14 day standard amphotericin B-based regimens . A non-inferiority design has been chosen as the primary aim of this trial is to identify an alternative safe and easy to administer short-course L-AmB treatment regimen that can be implemented in the settings where giving standard 7-14 day courses of amphotericin B deoxycholate-based treatment is difficult or impossible due to the need for daily intravenous infusions, close laboratory monitoring for the frequent drug induced toxicities, and intensive nursing and medical care. An efficacious single dose L-AmB treatment would also markedly facilitate CM therapy in settings

currently using standard courses of amphotericin B deoxycholate-based treatment, reducing the duration of hospitalisation and the associated risks(e.g. nosocomial sepsis) and costs. Ten-week mortality in our previous trials using amphotericin B deoxycholate-based regimens at the study sites has ranged from 28-41%[12,34], and is 30% with short-course high-dose L-AmB treatments in the recent phase-II study. We have therefore assumed a 35% 10-week mortality in both control and test groups. We want to show non-inferiority with a 10% margin (i.e. the upper margin of the one-sided 95% confidence interval of the difference in 10-week mortality between the two arms does not exceed 10%). The 10% margin has been chosen to ensure that only clinically unimportant differences are deemed non-inferior, and is in keeping with conventional practice. Using a one-sided a = 0.05 and 90% power, gives a sample size of 390 per arm (Figure 4). If the 10-week mortality is increased to 40% the equivalent sample size is 412 per arm. Making a conservative allowance for withdrawals and losses to follow-up of up to 8% (losses range from 2-4% in our trials), or a higher than anticipated mortality rate, we plan to enrol 425 patients per arm. Thus, we will randomise a total of ***850 patients.*** This will be the largest CM treatment trial conducted in Africa.

It is plausible that the intervention arm will lead to a greater reduction in mortality than the control arm through reduced toxicity or through another mechanism. In order to allow for this possibility, we have chosen to include superiority of the intervention as a secondary endpoint of the trial.

9.4 INTERIM MONITORING & ANALYSES

An IDMC Charter will be drawn up that describes the membership of the IDMC, relationships with other committees, terms of reference, and decision-making processes (with a description of stopping rules and/or guidelines, if any). One analysis will be done at the end of the trial. There will be no formal interim analyses. Regular data and safety monitoring will be done by the IDMC.

9.5 ANALYSIS PLAN (BRIEF)

Statistical analyses will be detailed in the statistical analysis plan. Briefly:

Analyses populations: 1) Intention-to-treat (ITT) population: all randomised subjects with valid informed consent; 2) Per-protocol (PP) population: a subset of the ITT population - subjects with major protocol deviations will be excluded from this PP population. 3) Safety population - a subset of the ITT population, consisting of all randomised subjects who receive at least one dose of study drug. 4) Pharmacokinetic (PK) population - a subset of the ITT population, consisting of all randomised subjects who receive at least one dose of study drug and have a PK profile. Primary analyses of efficacy will be based on the ITT population.

*The primary endpoint analysis**:*** The primary analysis will compare the treatment groups in terms of the proportion of participants who have died from any cause within 10 weeks. A generalised linear model (GLM) will be used. In this model, treatment group will be the sole predictor, using an identity-link function and binomial distribution to calculate an estimate for the (unadjusted) risk difference the treatment groups. If the upper limit of the one-sided 95% confidence interval (CI) for the risk difference falls below the non-inferiority margin of 10%, non-inferiority will be declared.

Secondary analyses.
We will conduct further analyses of the primary endpoint and analyse the secondary endpoints. These analyses will be done using a 5% two-sided significance level (i.e. to test for superiority).

Analyses of survival data will be conducted using unadjusted Cox regression analysis to calculate the hazard ratio (HR) and 95% CI between the treatment groups. Kaplan-Meier survival curves, and

survival percentages at 2, 4 and 10 weeks, will also be calculated by treatment group and overall. A log-rank test will be done to compare the survival curves between the treatment groups.

Proportions will be analysed using GLMs with treatment group as the sole predictor. The point estimate of the treatment effect with two-sided 95% CI will be derived.

For the analyses of cryptococcal clearance rates, we will perform linear regression of $\log_{10}$ CFU against time for each patient. The means rates of decrease in $\log_{10}$ CFU/ml/day will be compared between the two groups. We will calculate the difference in means together with the 95% CI. Rates of CFU decline will also be modelled using General Estimating Equations (GEE) methods.

Sensitivity analyses of the primary endpoint making different assumptions for the losses to follow-up will be conducted. Covariate-adjusted analyses for the primary endpoint will be conducted by adding pre-specified covariates into the GLM model to derive the adjusted risk difference and the upper limit of one-sided 95% CI. Imputation for baseline missing covariates will be made for the covariate-adjusted analysis.

Stratified analyses: Analyses stratified by ART status and other pre-defined baseline variables, such as clinical stage, will be performed. The other baseline variables will be defined in the analytical plan.

A full definition and explanation of all primary, secondary and stratified statistical analyses will be included in the statistical analysis plan.

PK-PD analysis: Plasma samples will be collected to describe the PK of liposomal amphotericin B, and enable the PK-PD of L-AmB for CM and the impact of PK variability on outcome to be determined. The serum PK of fluconazole will also be described. A portion of the CSF sample obtained for quantitative counts will be reserved to measure fluconazole concentrations, and thereby estimate the extent of penetration of fluconazole into the CSF. We will not attempt to measure concentrations of amphotericin B in CSF, as the drug is concentrated in the meninges and cannot be reliably detected in CSF. The PK-PD data will be modelled using a population methodology, using the program Pmetrics.

Safety: The safety analysis will be descriptive and the frequency and proportions of patients suffering clinical and laboratory-defined side effects (DAIDS grade III/IV adverse events) will be generated by treatment arms.

Economic analysis: An economic analysis will be conducted to provide evidence for the cost-effectiveness of short-course L-AmB treatment. The objective of the economic analysis is to estimate the cost consequences and the cost-effectiveness of short-course L-AmB treatment, compared to current care. Both societal and healthcare perspectives are chosen, and health service patient costs including household costs, treatment cost and hospitalisations in both arms will be compared over the trial period in a probabilistic approach, using Monte Carlo bootstrapping methods in STATA, @Risk software, and TreeAge. In the country-specific cost-consequence analyses, the societal and health service costs will be compared, and used along with the trial-wide primary endpoint data to perform cost-effectiveness modeling using a decision-tree model for each country with historical data as comparison.

ANCILLARY STUDIES

See Section 6.5. PK/PD studies and cost-effectiveness studies will be performed as outlined in this protocol. Cryptococcal isolates will be saved and shared with members of the cryptococcal research community for ongoing phenotypic and molecular epidemiology Blood and CSF samples will be saved for ongoing studies examining the phenotype of the immune response to Cryptococcus, and the pathophysiology of immune reconstitution inflammatory syndromes. Blood will also be used to determine how polymorphisms in immune response genes relate to susceptibility to and outcomes from cryptococcal meningitis. Any samples that require shipment will be handled in accordance with the local guidelines.

REGULATORY & ETHICAL ISSUES

11.1 COMPLIANCE

11.1.1 REGULATORY COMPLIANCE

The trial complies with the principles of the Declaration of Helsinki: as amended by *59th WMA General Assembly, Seoul, Korea, October 2008*.

It will also be conducted in compliance with the approved protocol, the principles of Good Clinical Practice (GCP) and applicable local laws and guidelines

11.1.2 SITE COMPLIANCE

The site will comply with the above and international sites will comply with the principles of GCP as laid down by the ICH topic E6 (Note for Guidance on GCP) and applicable national regulations. An agreement will be in place between the site and the co-ordinating centre, setting out respective roles and responsibilities (see Section 13 - Finance).

The site will inform the co-ordinating centre as soon as they are aware of a possible serious breach of compliance, so that the co-ordinating centre can report this breach if necessary within 7 days as per the UK regulatory requirements. For the purposes of this regulation, a 'serious breach' is one that is likely to affect to a significant degree:

- The safety or physical or mental integrity of the subjects in the trial, or
- The scientific value of the trial

11.1.3 DATA COLLECTION & RETENTION

eCRF data collected and validated using the EDC will be stored in an electronic database that is protected using a scheme of authentication and encryption (see below). Paper documents, such as clinical notes and administrative documentation should be kept in a secure location (for example, locked filing cabinets in a room with restricted access) and held for 5 years after the end of the trial. During this period, all data should be accessible to the competent or equivalent authorities, the Sponsor, (and other relevant parties) with suitable notice. The data may be subject to an audit by the competent authorities.

11.1.4 PROTECTION OF ELECTRONICALLY STORED SUBJECT DATA

Security of electronic records and data is a significant concern. All components of the distributed data systems will use authentication and encryption to render electronically stored subject identity and personal health information unusable, unreadable, or indecipherable to unauthorized individuals for “data in use” (e.g. data being analyzed by study investigators), “data in motion” [e.g. data being transferred between data entry points and the Data Management Center (DMC)] and “data at rest” (e.g. data in storage at DMC).

Full Drive Encryption will be implemented at the hardware layer of all devices storing protected health information. A three-factor scheme will be used to authenticate users through the hardware layer to the application layer where personal health information is available. The applications will have user profiles to control access to certain data and reports. The application and database layers will use a combination of hashing and encryption for sensitive and personal data. Mobile devices and the staff operating them will not be equipped with the encryption keys to decrypt select sensitive data fields.

11.2 ETHICAL CONDUCT OF THE STUDY

The Chief Investigators have overall responsibility for the ethical conduct of the trial.

11.2.1 ETHICAL CONSIDERATIONS

The patients or their family / guardians if unable to consent will, before being enrolled into the study, have the conditions of the study, as set out in the Patient Information Sheet explained to them. The information contained in the PIS will be translated into the appropriate local languages at each centre. Literate patients will be asked to read the PIS and illiterate patients will have the contents explained to them by the Local Investigator or Research Nurse. The patient will have the opportunity to discuss the PIS. Once the person taking consent is satisfied that the patient has understood the content of the PIS and Consent Form, the patient will be asked to sign the consent form. Original signed consent forms must be kept by the investigator and documented in the electronic case report form eCRF, a copy given to the participant or family and a copy placed in the participant's medical notes.

11.2.2 ETHICAL APPROVALS

The protocol will be submitted to the Investigational Studies Ethics Review Committee of the London School of Hygiene and Tropical Medicine. Before initiation of the trial at each clinical site, the protocol, all informed consent forms, and information materials to be given to the prospective participant will be submitted to each ethics committee for approval. Any further amendments will be submitted and approved by each ethics committee.

The rights of the participant to refuse to participate in the trial without giving a reason must be respected. After the participant has entered into the trial, the clinician must remain free to give alternative treatment to that specified in the protocol, at any stage, if he/she feels it to be in the best interest of the participant. The reason for doing so, however, should be recorded; the participant will remain within the trial for the purpose of follow-up and for data analysis by the treatment option to which they have been allocated. Similarly, the participant must remain free to change their mind at any time about the protocol treatment and trial follow-up without giving a reason and without prejudicing his/her further treatment.

11.3 COMPETENT AUTHORITY APPROVALS

This protocol will be reviewed by/submitted to the national competent or equivalent authority, as appropriate in each country where the trial will be run.

In countries where L-AmB is not registered, regulatory approvals/CTA will be sought from the country specific regulatory authorities.

The progress of the trial and safety issues will be reported to the competent authority, regulatory agency or equivalent in accordance with local requirements and practices in a timely manner.

Safety reports, including expedited reporting and SUSARS will be submitted to the competent authority in accordance with each authority's requirements in a timely manner.

11.4 OTHER APPROVALS

The protocol will be submitted by those delegated to do so to the relevant R&D department of each participating site or to other local departments for approval as required in each country. A copy of the

local R&D approval (or other relevant approval as above) and of the PIS and Consent Form on local headed paper should be forwarded to the co-ordinating centre before patients are entered.

INDEMNITY

The sponsor of the trial is the London School of Hygiene and Tropical Medicine and as such provides indemnity for the trial. All personnel involved in the trial will be expected to be indemnified by their employing authority. Local insurance will be taken out where local regulations require this.

FINANCE

The study is jointly funded through the European Developing Countries Clinical Trials Partnership (EDCTP) and Wellcome Trust / Medical Research Council (UK) / UKAID Joint Global Health Trials.
See Study Budget for further details.

OVERSIGHT & TRIAL COMMITTEES

There are a number of committees involved with the oversight of the trial. These committees are detailed below.

14.1 TRIAL MANAGEMENT GROUP (TMG)

A Trial Management Group (TMG) will be formed comprising the Chief Investigators, supported by lead investigators (clinical and non-clinical) as needed and members of the co-ordinating centre. The TMG will be responsible for the day-to-day running and management of the trial. It will meet approximately three times a year at least one of which will be in-person. The full details can be found in the TMG Charter.

14.2 TRIAL STEERING COMMITTEE (TSC)

The Trial Steering Committee (TSC) has membership from the TMG (Dr Joseph Jarvis, Professor Tom Harrison, Professor Shabbar Jaffar, Professor David Lalloo, Professor Graeme Meintjes, Professor Mina Hosseinipour, Dr David Boulware, Dr David Meya, Professor Olivier Lortholary – three as voting memebers (Jarvis, Harrison, Jaffar) and the rest as observer) plus three independent voting members, including the Chair. The role of the TSC is to provide overall supervision for the trial and provide advice through its independent Chair. The ultimate decision for the continuation of the trial lies with the TSC. Further details of TSC functioning are presented in the TSC Charter. The independent academic chair will be Professor John Perfect, Duke University, with 3 additional independent members; Dr Saidi Kapiga (Head of MITU, Mwanza, Tanzania), Dr Douglas Wilson (Edendale Hospital, South Africa) and Dr Andrew Kambugu (Head of Research, Infectious Diseases Institute, Uganda). The EDCTP will appoint a TSC member with observer status. The lead clinician and site PIs will be attend as observers. This steering committee will meet at the beginning and then annually and will be responsible for the overall direction of the research.

14.3 INDEPENDENT DATA MONITORING COMMITTEE (IDMC)

An Independent Data Monitoring Committee (IDMC) will be formed. The IDMC will be the only group who sees the confidential, accumulating data for the trial. Reports to the IDMC will be produced by the trial statistician, supported by the co-ordinating centre statisticians. The IDMC will meet within 6 months of the trial opening; the frequency of meetings will be dictated in the IDMC charter. The IDMC will consider data using the statistical analysis plan (see Section 9.5 - Analysis Plan (Brief)) and will advise the TSC. The IDMC can recommend premature closure or reporting of the trial, or that recruitment to any research arm be discontinued.

Further details of IDMC functioning, and the procedures for analysis and monitoring, are provided in the IDMC Charter. The committee will comprise Prof Andrew Nunn, UCL, UK (Chair, Statistician), Dr Rob Peck, Cornell University (Clinician/trialist), Dr Sayoki Mfinanga, Muhimbili Medical Research Centre, National Institute for Medical Research, United Republic of Tanzania (Clinician/trialist) and Dr William Powderly, Washington University in St. Louis (Clinician/trialist).

PUBLICATION

The results from the different centres will be analysed together and published as soon as possible. Individual clinicians must not publish data concerning their patients that are directly relevant to questions posed by the study until the Trial Management Group has published its report.
The Trial Management Group will form the basis of the Writing Committee and will advise on the nature of the publication. The names of all investigators who have contributed will be included in the authorship of any publication. The authorship policy will be agreed before any publication and will be inclusive and decided in a fair transparent manner. The members of the TSC and IDMC will be listed with their affiliations in the Acknowledgements/Appendix of the main publication. The funders will have no role in the decision to publish or the content of the publication.

PROTOCOL AMENDMENTS

V 1.0 22/06/2017

V 1.1 29/06/2017

1. P3, SAE Reporting Box: SAEs must be reported within 24 hours – changed from 48

2. P3, Coordinating Site Staff Table: Email address updated for Dr Timothée Boyer-Chammard

3. P4, Co-Investigators Table: Contact details added for Dr Tom Crede and Dr Charlotte Schutz

4. P45, 7.3.1.E: SAEs must be reported within 24 hours - changed from 48

5. P47, 8.3: Addition of 'The study may be subject to audit by the London School of Hygiene & Tropical Medicine under their remit as sponsor, the Study Coordination Centre and other regulatory bodies to ensure adherence to GCP.'

6. P47, 8.3.2: Addition of 'Consent forms will be stored under the supervision of each local PI in a secured office and accessible to trial staff only. The database does not hold personal details as participants are identified by their study number throughout the trial.'

V 1.2 24/08/2017

1. P3, Randomisation system helpdesk contacts added.

2. P5, Coordinating Site Staff Table: details included for Dr Alexandre Alanio.

3. P8, Summary of Trial table updated with ISRCTN, Sponsor and LSHTM REC references numbers.

4. P8, addition of *Following the recently closed ACTA trial, if the new standard becomes 7 days of amphotericin rather than 14 days we will amend the control arm accordingly.

5. P8, addition of 'Single-dose high-dose L-AmB given with high dose fluconazole and flucytosine will be non-inferior to standard daily-dosed Amphotericin B deoxycholate based induction therapy with flucytosine for the treatment of HIV-associated cryptococcal meningitis in averting all-cause mortality.'

6. P8, removal of 'Short-course high-dose L-AmB given with high dose fluconazole and flucytosine will be non-inferior to 2 weeks daily-dosed Amphotericin B deoxycholate based induction therapy with flucytosine for the treatment of HIV-associated cryptococcal meningitis in averting all-cause mortality.'

7. P10, Schema updated to show 'day for 14 days +'.

8. P12, addition of '*Results from the recently completed ACTA phase III randomised controlled trial have shown that 7-days of amphotericin B deoxycholate is superior to 14-days (HR 0.56 (0.35-0.91)). These findings were presented at the International AIDS Society Conference on HIV Science, Paris, July 2017 and are awaiting peer-review. Following peer review if 7-days is confirmed as the new standard an amendment will be submitted to switch the control arm to

treatment with 7-days of amphotericin B deoxycholate followed by 7-days of fluconazole 1200mg/d.'

9. P19, addition of 'This theory has been supported by the recently completed Advancing Cryptococcal Meningitis Treatment for Africa (ACTA) trial which demonstrated that 1-week of amphotericin B deoxycholate is non-inferior to 2-weeks of treatment in averting all-cause mortality in patients with first episode of HIV-associated CM[53].

10. P21, addition of 'superior compared with those achieved with 14-day courses (HR 0.56 (0.35-0.91)). These findings were presented at the International AIDS Society Conference on HIV Science, Paris, July 2017 and are awaiting peer-review. Following peer-review if 7-days is confirmed as the new standard an amendment will be submitted to switch the control arm to treatment with 7-days of amphotericin B deoxycholate followed by 7-days of fluconazole 1200mg/d. The ACTA study also confirmed that flucytosine (5FC) is a significantly superior partner drug for amphotericin B based treatments compared with fluconazole, leading to a substantial mortality reduction of 32% (95%CI 16 - 55%, p=0.002)[53].

11. P21, removal of 'statistically non-inferior (at a 10% non-inferiority margin at 2 weeks) compared with those achieved with 14 day courses. The study also confirmed that flucytosine (5FC) is a significantly superior partner drug for amphotericin B based treatments compared with fluconazole, leading to a substantial mortality reduction of 32% (95%CI 16 - 55%, p=0.002) (Harrison, ACTA Trial, Unpublished).'

12. P33, addition of '*Results from the recently completed ACTA phase III randomised controlled trial have shown that 7-days of amphotericin B deoxycholate is superior to 14-days (HR 0.56 (0.35-0.91)). These findings were presented at the International AIDS Society Conference on HIV Science, Paris, July 2017 and are awaiting peer-review. Following peer review if 7-days is confirmed as the new standard an amendment will be submitted to switch the control arm to treatment with 7-days of amphotericin B deoxycholate followed by 7-days of fluconazole 1200mg/d.'

13. P59, addition of Drs Wilson and Kambugu as TSC members.

14. P65, addition of reference 53 '. Molloy, S; Kanyama, C; Heyderman, R; Loyse, A; Kouanfack, C; Chanda, D; Mfinanga, S; Temfack, E; Lakhi, S; Lesikari, S; et al. (2017) A randomized controlled trial for the treatment of HIV-associated cryptococcal meningitis in Africa: oral fluconazole plus flucytosine or one week amphotericin-based therapy versus two weeks amphotericin-based therapy. The ACTA Trial. (in draft)

V 2.0 18/10/2017

1. P.8, Removal of '*Following the recently closed ACTA trial, if the new standard becomes 7 days of amphotericin rather than 14 days we will amend the control arm accordingly.' Treatment arms updated to 1) L-AmB 10 mg/kg day 1 (single dose) with flucytosine 100mg/kg/d and fluconazole 1200mg/day for 14-days. 2) Amphotericin-B deoxycholate 1mg/kg/d for 7-days with flucytosine 100mg/kg/d(standard dose "control arm") followed by 7-days of fluconazole 1200mg/day.

2. P.8 Study hypothesis changed from 'Single-dose high-dose L-AmB given with high dose fluconazole and flucytosine will be non-inferior to standard daily-dosed Amphotericin B deoxycholate based induction therapy with flucytosine for the treatment of HIV-associated cryptococcal meningitis in averting all-cause mortality.' to 'Single high-dose L-AmB given with

14-days of high-dose fluconazole will be non-inferior to 7-days of standard dosed Amphotericin B deoxycholate followed by 7-days of high dose fluconazole induction therapy, both given with flucytosine, for the treatment of HIV-associated cryptococcal meningitis in averting all-cause mortality.'

3. P.10, Updated treatment regimen in Control box.

4. P.11, 'Current guidelines recommend treatment with amphotericin B deoxycholate which requires 14 days of intravenous infusions given in hospital…..' changed to 'Current guidelines recommend treatment with amphotericin B deoxycholate which requires 7 days of intravenous infusions given in hospital….'

5. P.11, removal of conventional.

6. P.11, 'standard 14 days' changed to '7 days'.

7. P.11, Trial interventions changed from '1. L-AmB 10 mg/kg day 1 ("single dose") 2. Amphotericin B deoxycholate 1 mg/kg/d for 14 days (standard dose, "control arm")* Patients in the single dose arm will receive also fluconazole 1200mg/d for the first 14 days.'to '1. L-AmB 10 mg/kg day 1 ("single dose") given with fluconazole 1200mg/day plus flucytosine 100mg/kg/d for 14-days. 2. Amphotericin B deoxycholate 1 mg/kg/d for 7-days given with flucytosine 100mg/kg/d (standard dose, "control arm") followed by fluconazole 1200mg/day for 7-days.'

8. P.12, 'Standard induction therapy for CM is 14 days. In the control arm this will consist of daily amphotericin B deoxycholate infusions plus oral flucytosine 100mg/kg/day. In the short-course arm this will consist of a single dose of L-AmB plus high dose (1200mg) oral fluconazole and oral flucytosine 100mg/kg/day.' changed to 'Standard induction therapy for CM is 14 days. In the control arm this will consist of 7-days of daily amphotericin B deoxycholate infusions plus oral flucytosine 100mg/kg/day followed by 7-days of high-dose (1200mg/day) oral fluconazole. In the short-course arm this will consist of a single dose of L-AmB plus 14-days of high dose (1200mg/day) oral fluconazole and oral flucytosine 100mg/kg/day.'

9. P.12, Removal of '*Results from the recently completed ACTA phase III randomised controlled trial have shown that 7-days of amphotericin B deoxycholate is superior to 14-days (HR 0.56 (0.35-0.91)). These findings were presented at the International AIDS Society Conference on HIV Science, Paris, July 2017 and are awaiting peer-review. Following peer review if 7-days is confirmed as the new standard an amendment will be submitted to switch the control arm to treatment with 7-days of amphotericin B deoxycholate followed by 7-days of fluconazole 1200mg/d.'

10. P.20, 'Amphotericin B deoxycholate-based therapy requires hospitalization for 14 days' changed to 'Amphotericin B deoxycholate-based therapy requires hospitalization for at least 7-14 days'.

11. P.20, 'Current guidelines recommend 2-week courses of amphotericin B deoxycholate-based treatment.' changed to 'International treatment guidelines have previously recommended 2-week courses of amphotericin B deoxycholate-based treatment.'

12. P.20, 'This theory has been supported by the recently completed Advancing Cryptococcal Meningitis Treatment for Africa (ACTA) trial …..' changed to 'This theory has been furthered

by the recently completed clinical-endpoint Advancing Cryptococcal Meningitis Treatment for Africa (ACTA) trial…..'

13. P21, '14 days ' changed to 7 days'.

14. P.21, 'Current treatment guidelines recommend 2-week courses of amphotericin B deoxycholate based treatment' changed to 'Until recently international treatment guidelines recommended 2-week courses of amphotericin B deoxycholate based treatment'.

15. P.21, 'These findings were presented at the International AIDS Society Conference on HIV Science, Paris, July 2017 and are awaiting peer-review. Following peer-review if 7-days is confirmed as the new standard an amendment will be submitted to switch the control arm to treatment with 7-days of amphotericin B deoxycholate followed by 7-days of fluconazole 1200mg/d.' changed to 'These findings were presented at the International AIDS Society Conference on HIV Science, Paris, July 2017 and have subsequently been incorporated into updated World Health Organisation guidelines (in press)'.

16. P.33, '1. L-AmB 10 mg/kg day 1 (single dose)
    2. Amphotericin B deoxycholate 1 mg/kg/d for 14-days (standard dose, "control arm")*
    Patients in the single dose arm will receive fluconazole 1200mg/d po for the first 14 days. All patients also receive flucytosine 100mg/kg/d po for first 2 weeks, and then fluconazole 800 mg/d po to 10 weeks (provision will be ensured throughout the trial), and 200 mg/d thereafter (as per national guidelines at the site). ART will be commenced 4 – 6 weeks after initiation of antifungal therapy.

    Changed to '1. L-AmB 10 mg/kg day 1 (single dose) given with fluconazole 1200mg/day plus flucytosine 100mg/kg/d for 14-days
    2. Amphotericin B deoxycholate 1 mg/kg/d for 7-days given with flucytosine 100mg/kg/d followed by fluconazole 1200mg/day for 7-days (standard dose, "control arm")
    All patients receive fluconazole 800 mg/d to 10 weeks (provision will be ensured throughout the trial), and 200 mg/d thereafter (as per national guidelines at the site). ART will be commenced 4 – 6 weeks after initiation of antifungal therapy.'

17. P.33, Removal of '*Results from the recently completed ACTA phase III randomised controlled trial have shown that 7-days of amphotericin B deoxycholate is superior to 14-days (HR 0.56 (0.35-0.91)). These findings were presented at the International AIDS Society Conference on HIV Science, Paris, July 2017 and are awaiting peer-review. Following peer review if 7-days is confirmed as the new standard an amendment will be submitted to switch the control arm to treatment with 7-days of amphotericin B deoxycholate followed by 7-days of fluconazole 1200mg/d.'

    P.33, 'Initial intensive phase (first 2 weeks): Patients will be admitted to hospital for the initial intensive phase of treatment, with all patients managed as inpatients for at least 7 days. Individuals in the short-course L-AmB arm may be discharged after day 7 and prior to day 14….' Changed to 'Initial intensive phase (first 2 weeks): Patients will be admitted to hospital for the initial intensive phase of treatment, with all patients managed as inpatients for at least 7-days. Individuals may be discharged after day 7 and prior to day 14…...'

18. P.34, 'The initial two weeks of treatment will be given in a directly observed hospital setting, or under close outpatient supervision during the second week of the short-course L-AmB arm, ensuring compliance to the trial intervention.' changed to 'The first 7-days of the two week

induction treatment will be given in a directly observed hospital setting. Patients may be discharged from day 7 at which point they will remain under close outpatient supervision during the second week, ensuring compliance to the trial intervention.'.

19. P.37 'In the single dose L-AmB arm patients may be discharged from day 7, in which case they will receive close outpatient follow-up….' changed to 'Patients may be discharged from day 7, in which case they will receive close outpatient follow-up….'.

20. P.49, 14 days changed to 7-14 days.

V 2.1 07/11/2017

1. P.6, Addition of Dr Conrad Muzoora as Co-Investigator in Uganda.

2. P.8, Removal of Harare Central hospital.

3. P.12, 'Patients who have been exposed to ART will be followed-up with repeat viral loads and resistance testing, as per the standard operating procedure for this group, and data collected about rates or treatment failure and drug resistance.' changed to 'Patients who have been exposed to ART will be followed-up with repeat viral loads and resistance testing, as per the standard procedures at the sites, and data collected about rates of treatment failure and drug resistance.

4. P.19, 'This theory has been furthered by the recently completed clinical-endpoint Advancing Cryptococcal Meningitis Treatment for Africa (ACTA) trial which demonstrated that 1-week of amphotericin B deoxycholate is superior to 2-weeks of treatment in averting all-cause mortality in patients with first episode of HIV-associated CM (HR 0.56 (0.35-0.91)[53]. As a consequence of these findings, guidelines now recommend a 1 week course of amphotericin B deoxycholate-based treatment.' changed to 'This theory has been furthered by the recently completed clinical-endpoint Advancing Cryptococcal Meningitis Treatment for Africa (ACTA) trial which demonstrated that 1-week of amphotericin B deoxycholate with flucytosine is superior to 2-weeks of treatment in averting all-cause mortality in patients with first episode of HIV-associated CM (HR 0.56 (0.35-0.91)[53]. As a consequence of these findings, guidelines now recommend a 1 week course of amphotericin B deoxycholate-based treatment with flucytosine.

5. P.21, Addition of 'with flucytosine'.

6. P.27, Removal of Harare Central hospital.

7. P29, 'The site should conduct the trial in compliance with the protocol as agreed by the Sponsor and, if required, by the regulatory authority(ies), and which was given favourable opinion by the REC and/or IRB.' changed to 'The site should conduct the trial in compliance with the protocol as agreed by the Sponsor and, if required, by the regulatory authority(ies), and which has been approved by the REC and/or IRB.

8. P.34, Removal of link referencing solution and insertion of link referencing to flucytosine tablets.

REFERENCES

1. Braitstein P, Brinkhof MW, Dabis F, et al. Mortality of HIV-1-infected patients in the first year of antiretroviral therapy: comparison between low-income and high-income countries. *Lancet* 2006; **367**(9513): 817-24.
2. Lawn S, Harries A, Anglaret X, Myer L, Wood R. Early Mortality among adults accessing antiretroviral treatment programmes in sub-Saharan Africa. *AIDS (London, England)* 2008; **22**(15): 1897-908.
3. Amuron B, Namara G, Birungi J, et al. Mortality and loss-to-follow-up during the pre-treatment period in an antiretroviral therapy programme under normal health service conditions in Uganda. *BMC Public Health* 2009; **9**: 290.
4. Gupta A, Nadkarni G, Yang WT, et al. Early mortality in adults initiating antiretroviral therapy (ART) in low- and middle-income countries (LMIC): a systematic review and meta-analysis. *PLoS ONE* 2011; **6**(12): e28691.
5. Park BJ, Wannemuehler KA, Marston BJ, Govender N, Pappas PG, Chiller TM. Estimation of the current global burden of cryptococcal meningitis among persons living with HIV/AIDS. *AIDS (London, England)* 2009; **23**(4): 525-30.
6. Jarvis JN, Boulle A, Loyse A, et al. High ongoing burden of cryptococcal disease in Africa despite antiretroviral roll out. *AIDS (London, England)* 2009; **23**: 1181–5.
7. Jarvis JN, Meintjes G, Williams A, Brown Y, Crede T, Harrison TS. Adult meningitis in a setting of high HIV and TB prevalence: findings from 4961 suspected cases. *BMC Infect Dis* 2010; **10**(1): 67.
8. Wall EC, Everett DB, Mukaka M, et al. Bacterial meningitis in Malawian adults, adolescents, and children during the era of antiretroviral scale-up and Haemophilus influenzae type b vaccination, 2000-2012. *Clin Infect Dis* 2014; **58**(10): e137-45.
9. Jarvis JN, Harrison TS. Forgotten but not gone: HIV-associated cryptococcal meningitis. *The Lancet infectious diseases* 2016.
10. Jarvis JN, Bicanic T, Loyse A, et al. Determinants of mortality in a combined cohort of 501 patients with HIV-associated Cryptococcal meningitis: implications for improving outcomes. *Clin Infect Dis* 2014; **58**(5): 736-45.
11. Bicanic T, Wood R, Meintjes G, et al. High-dose amphotericin B with flucytosine for the treatment of cryptococcal meningitis in HIV-infected patients: a randomized trial. *Clin Infect Dis* 2008; **47**(1): 123-30.
12. Jarvis JN, Meintjes G, Rebe K, et al. Adjunctive interferon-gamma immunotherapy for the treatment of HIV-associated cryptococcal meningitis: a randomized controlled trial. *AIDS (London, England)* 2012; **26**(9): 1105-13.
13. Longley N, Muzoora C, Taseera K, et al. Dose response effect of high-dose fluconazole for HIV-associated cryptococcal meningitis in southwestern Uganda. *Clin Infect Dis* 2008; **47**(12): 1556-61.
14. Jackson A, Nussbaum J, Phulusa J, et al. A Phase II Randomised Controlled Trial Adding Oral Flucytosine to High Dose Fluconazole, with Short-course Amphotericin B, for Cryptococcal Meningitis in Malawi. *AIDS (London, England)* 2012; **26**(11): 1363-70.
15. Muzoora CK, Kabanda T, Ortu G, et al. Short course amphotericin B with high dose fluconazole for HIV-associated cryptococcal meningitis. *The Journal of infection* 2011; **64**(1): 76-81.
16. Livermore J, Howard SJ, Sharp AD, et al. Efficacy of an abbreviated induction regimen of amphotericin B deoxycholate for cryptococcal meningoencephalitis: 3 days of therapy is equivalent to 14 days. *MBio* 2013; **5**(1): e00725-13.
17. Hope WW, Goodwin J, Felton TW, Ellis M, Stevens DA. Population pharmacokinetics of conventional and intermittent dosing of liposomal amphotericin B in adults: a first critical step for rational design of innovative regimens. *Antimicrobial agents and chemotherapy* 2012; **56**(10): 5303-8.

18. O'Connor L, Livermore J, Sharp AD, et al. Pharmacodynamics of liposomal amphotericin B and flucytosine for cryptococcal meningoencephalitis: safe and effective regimens for immunocompromised patients. *The Journal of infectious diseases* 2013; **208**(2): 351-61.
19. Gubbins PO, Amsden JR, McConnell SA, Anaissie EJ. Pharmacokinetics and buccal mucosal concentrations of a 15 milligram per kilogram of body weight total dose of liposomal amphotericin B administered as a single dose (15 mg/kg), weekly dose (7.5 mg/kg), or daily dose (1 mg/kg) in peripheral stem cell transplant patients. *Antimicrobial agents and chemotherapy* 2009; **53**(9): 3664-74.
20. Mehta P, Vinks A, Filipovich A, et al. High-dose weekly AmBisome antifungal prophylaxis in pediatric patients undergoing hematopoietic stem cell transplantation: a pharmacokinetic study. *Biol Blood Marrow Transplant* 2006; **12**(2): 235-40.
21. Vogelsinger H, Weiler S, Djanani A, et al. Amphotericin B tissue distribution in autopsy material after treatment with liposomal amphotericin B and amphotericin B colloidal dispersion. *The Journal of antimicrobial chemotherapy* 2006; **57**(6): 1153-60.
22. Sundar S, Chakravarty J, Agarwal D, Rai M, Murray HW. Single-dose liposomal amphotericin B for visceral leishmaniasis in India. *The New England journal of medicine* 2010; **362**(6): 504-12.
23. Cornely OA, Maertens J, Bresnik M, et al. Liposomal amphotericin B as initial therapy for invasive mold infection: a randomized trial comparing a high-loading dose regimen with standard dosing (AmBiLoad trial). *Clin Infect Dis* 2007; **44**(10): 1289-97.
24. Hamill RJ, Sobel JD, El-Sadr W, et al. Comparison of 2 doses of liposomal amphotericin B and conventional amphotericin B deoxycholate for treatment of AIDS-associated acute cryptococcal meningitis: a randomized, double-blind clinical trial of efficacy and safety. *Clin Infect Dis* 2010; **51**(2): 225-32.
25. Bicanic T, Bottomley C, Loyse A, et al. Toxicity of Amphotericin B Deoxycholate-Based Induction Therapy in Patients with HIV-Associated Cryptococcal Meningitis. *Antimicrobial agents and chemotherapy* 2015; **59**(12): 7224-31.
26. Perfect JR, Dismukes WE, Dromer F, et al. Clinical practice guidelines for the management of cryptococcal disease: 2010 update by the infectious diseases society of america. *Clin Infect Dis* 2010; **50**(3): 291-322.
27. Nelson M, Dockrell D, Edwards S, et al. British HIV Association and British Infection Association guidelines for the treatment of opportunistic infection in HIV-seropositive individuals 2011. *HIV medicine* 2011; **12 Suppl 2**: 1-140.
28. Molefi M, Chofle AA, Molloy SF, et al. AMBITION-cm: intermittent high dose AmBisome on a high dose fluconazole backbone for cryptococcal meningitis induction therapy in sub-Saharan Africa: study protocol for a randomized controlled trial. *Trials* 2015; **16**: 276.
29. Jarvis JN, Leeme T, Chofle AA, et al. Ambition-CM: High-dose Liposomal Amphotericin for HIV-related Cryptococcal Meningitis. Conference on Retroviruses and Opportunistic Infections 2017. Seattle, WA.; 2017.
30. Day JN, Chau TT, Wolbers M, et al. Combination antifungal therapy for cryptococcal meningitis. *The New England journal of medicine* 2013; **368**(14): 1291-302.
31. Pappas PG, Chetchotisakd P, Larsen RA, et al. A phase II randomized trial of amphotericin B alone or combined with fluconazole in the treatment of HIV-associated cryptococcal meningitis. *Clin Infect Dis* 2009; **48**(12): 1775-83.
32. Bicanic T, Muzoora C, Brouwer AE, et al. Independent association between rate of clearance of infection and clinical outcome of HIV-associated cryptococcal meningitis: analysis of a combined cohort of 262 patients. *Clin Infect Dis* 2009; **49**(5): 702-9.
33. Bisson GP, Molefi M, Bellamy S, et al. Early versus delayed antiretroviral therapy and cerebrospinal fluid fungal clearance in adults with HIV and cryptococcal meningitis. *Clin Infect Dis* 2013; **56**(8): 1165-73.
34. Beardsley J, Wolbers M, Kibengo FM, et al. Adjunctive Dexamethasone in HIV-Associated Cryptococcal Meningitis. *The New England journal of medicine* 2016; **374**: 542-54.

35. Boulware DR, Meya DB, Muzoora C, et al. Timing of antiretroviral therapy after diagnosis of cryptococcal meningitis. *The New England journal of medicine* 2014; **370**(26): 2487-98.

36. Rolfes MA, Hullsiek KH, Rhein J, et al. The effect of therapeutic lumbar punctures on acute mortality from cryptococcal meningitis. *Clin Infect Dis* 2014; **59**(11): 1607-14.

37. Nussbaum JC, Jackson A, Namarika D, et al. Combination flucytosine and high-dose fluconazole compared with fluconazole monotherapy for the treatment of cryptococcal meningitis: a randomized trial in Malawi. *Clin Infect Dis* 2009; **50**(3): 338-44.

38. Rothe C, Sloan DJ, Goodson P, et al. A prospective longitudinal study of the clinical outcomes from cryptococcal meningitis following treatment induction with 800 mg oral fluconazole in Blantyre, Malawi. *PLoS ONE* 2013; **8**(6): e67311.

39. Steele KT, Thakur R, Nthobatsang R, Steenhoff AP, Bisson GP. In-hospital mortality of HIV-infected cryptococcal meningitis patients with C. gattii and C. neoformans infection in Gaborone, Botswana. *Med Mycol* 2010; **48**(8): 1112-5.

40. Makadzange AT, Ndhlovu CE, Takarinda K, et al. Early versus delayed initiation of antiretroviral therapy for concurrent HIV infection and cryptococcal meningitis in sub-saharan Africa. *Clin Infect Dis* 2010; **50**(11): 1532-8.

41. Pac L, Horwitz MM, Namutebi AM, et al. Implementation and operational research: Integrated pre-antiretroviral therapy screening and treatment for tuberculosis and cryptococcal antigenemia. *Journal of acquired immune deficiency syndromes (1999)* 2015; **68**(5): e69-76.

42. Rhein J, Morawski BM, Hullsiek KH, et al. Efficacy of Adjunctive Sertraline for the Treatment of HIVAssociated Cryptococcal Meningitis: an open-label dose-ranging study. *Lancet Infectious Diseases* 2016; **TBC**.

43. Bisson GP, Nthobatsong R, Thakur R, et al. The use of HAART is associated with decreased risk of death during initial treatment of cryptococcal meningitis in adults in Botswana. *Journal of acquired immune deficiency syndromes (1999)* 2008; **49**(2): 227-9.

44. Gaskell KM, Rothe C, Gnanadurai R, et al. A prospective study of mortality from cryptococcal meningitis following treatment induction with 1200 mg oral fluconazole in Blantyre, Malawi. *PLoS ONE* 2014; **9**(11): e110285.

45. Govender NP, Meintjes G, Bicanic T, et al. Guideline for the prevention, diagnosis and management of cryptococcal meningitis among HIV-infected persons: 2013 update. *Southern African Journal of HIV Medicine* 2013; **14**(2): 76-86.

46. WHO Rapid advice: Diagnosis, prevention and management of cryptococcal disease in HIV-infected adults, adolescents and children. WHO 2011.

47. Brouwer AE, Rajanuwong A, Chierakul W, et al. Combination antifungal therapies for HIV-associated cryptococcal meningitis: a randomised trial. *Lancet* 2004; **363**(9423): 1764-7.

48. U.S. Department of Health and Human Services NIoH, National Institute of Allergy and Infectious Diseases, Division of AIDS. Division of AIDS (DAIDS) Table for Grading the Severity of Adult and Pediatric Adverse Events, Version 2.1. . 2017.

49. Jarvis JN, Casazza JP, Stone HH, et al. The phenotype of the Cryptococcus-specific CD4+ memory T-cell response is associated with disease severity and outcome in HIV-associated cryptococcal meningitis. *The Journal of infectious diseases* 2013; **207**(12): 1817-28.

50. Jarvis JN, Bicanic T, Loyse A, et al. Very Low Levels of 25-Hydroxyvitamin D Are Not Associated With Immunologic Changes or Clinical Outcome in South African Patients With HIV-Associated Cryptococcal Meningitis. *Clin Infect Dis* 2014.

51. Jarvis JN, Meintjes G, Bicanic T, et al. Cerebrospinal fluid cytokine profiles predict risk of early mortality and immune reconstitution inflammatory syndrome in HIV-associated cryptococcal meningitis. *PLoS Pathog* 2015; **11**(4): e1004754.

52. Beale MA, Sabiiti W, Robertson EJ, et al. Genotypic Diversity Is Associated with Clinical Outcome and Phenotype in Cryptococcal Meningitis across Southern Africa. *PLoS neglected tropical diseases* 2015; **9**(6): e0003847.

53. Molloy, S; Kanyama, C; Heyderman, R; Loyse, A; Kouanfack, C; Chanda, D; Mfinanga, S; Temfack, E; Lakhi, S; Lesikari, S; et al. (2017) A randomized controlled trial for the treatment of HIV-associated

cryptococcal meningitis in Africa: oral fluconazole plus flucytosine or one week amphotericin-based therapy versus two weeks amphotericin-based therapy. The ACTA Trial. (in draft)

APPENDICES

18.1 APPENDIX 1 - DIVISION OF AIDS TABLE FOR GRADING THE SEVERITY OF ADULT AND PEDIATRIC ADVERSE EVENTS
